# The Neural Bases of Directed and Spontaneous Mental State Attributions to Group Agents

**DOI:** 10.1371/journal.pone.0105341

**Published:** 2014-08-20

**Authors:** Adrianna C. Jenkins, David Dodell-Feder, Rebecca Saxe, Joshua Knobe

**Affiliations:** 1 Helen Wills Neuroscience Institute and Haas School of Business, University of California, Berkeley, California, United States of America; 2 Department of Psychology, Harvard University, Cambridge, Massachusetts, United States of America; 3 Department of Brain and Cognitive Sciences, Massachusetts Institute of Technology, Cambridge, Massachusetts, United States of America; 4 Program in Cognitive Science, Yale University, New Haven, Connecticut, United States of America; University of Medicine & Dentistry of NJ - New Jersey Medical School, United States of America

## Abstract

In daily life, perceivers often need to predict and interpret the behavior of group agents, such as corporations and governments. Although research has investigated how perceivers reason about individual members of particular groups, less is known about how perceivers reason about group agents themselves. The present studies investigate how perceivers understand group agents by investigating the extent to which understanding the ‘mind’ of the group as a whole shares important properties and processes with understanding the minds of individuals. Experiment 1 demonstrates that perceivers are sometimes willing to attribute a mental state to a group as a whole even when they are not willing to attribute that mental state to any of the individual members of the group, suggesting that perceivers can reason about the beliefs and desires of group agents over and above those of their individual members. Experiment 2 demonstrates that the degree of activation in brain regions associated with attributing mental states to individuals—i.e., brain regions associated with mentalizing or theory-of-mind, including the medial prefrontal cortex (MPFC), temporo-parietal junction (TPJ), and precuneus—does not distinguish individual from group targets, either when reading statements about those targets' mental states (directed) or when attributing mental states implicitly in order to predict their behavior (spontaneous). Together, these results help to illuminate the processes that support understanding group agents themselves.

## Introduction

In domains ranging from the economy to national security, large-scale decisions often involve judgments about the machinations of a group agent, such as a terrorist organization, government, or corporation. Sometimes, judgments about a group agent simply reduce to judgments about one or more of its individual members (for example, thinking about whether or not a *country* is hiding nuclear weapons may primarily involve consideration of that country's *leader*). However, people also sometimes appear to make judgments about a group by treating it as an entity in and of itself. Individuals assign moral blame and punishment to whole organizations [1], interpret laws by looking for the ‘intentions’ of the legislature [2], may get into financial trouble by reasoning about the ‘mind’ of the market [3], and, in a recent decision by the United States Supreme Court, extended rights typically granted to individuals to a corporation as a whole [4].

Although an abundance of research has investigated the effects of group membership on how people perceive and reason about the minds of individual people (for recent reviews, see [5]–[7], less is known about how perceivers reason about the ‘mind’ of a group agent itself [8]. To investigate this question, the present work uses a combination of behavioral and fMRI approaches to examine the extent to which understanding the ‘mind’ of the group as a whole shares important properties and processes with understanding the minds of individuals. Specifically, we ask (1) to what extent people sometimes reason about the beliefs and intentions of a group agent separately from those of the groups’ members and (2) to what extent brain regions associated with understanding individuals also support understanding group agents.

In order to predict or understand the behavior of a single individual, perceivers often appeal to that individual’s *mental states* (i.e., his or her thoughts, beliefs, intentions, desires, and feelings). This capacity to ascribe mental states to others—that is, to *mentalize* [9], [10] or engage *theory-of-mind* [11], [12]—reveals itself in the words perceivers use when talking about other people. For example, we can say that Dick *thought* he was aiming for a partridge and never *intended* to shoot his friend. Words like *think, believe, feel, intend, want*, and *plan* all refer to the inner contents of other minds, allowing perceivers to speak about the purported underlying causes of others’ behavior even as they diverge from that behavior itself [13], [14]. In turn, inferences about these internal causes guide moral decisions about how others should be treated, including the extent to which they deserve praise or punishment [15], [16].

Over the past two decades, an abundance of neuroimaging research has linked mentalizing or theory-of-mind to a consistent set of brain regions, including the medial prefrontal cortex (MPFC), temporo-parietal junction (TPJ), and precuneus/posterior cingulate, sometimes collectively called the ‘theory-of-mind network’. Using carefully controlled tasks that aim to isolate theory-of-mind, these regions show preferential engagement when people are thinking about humans versus other entities [17]–[24] and when people are thinking about humans' minds versus their other aspects, such as their physical attributes [21], [25]–[27]. Although much of this evidence has been correlational, recent work using TMS has demonstrated a causal role for the Right TPJ (RTPJ) in the use of mental state information for moral judgment [15], and research on individuals with damage to MPFC and TPJ has demonstrated a role for those regions in the ability to make inferences about others' mental states [28], [29].

Intriguingly, mental state words pervade perceivers' statements not only about individuals but also about groups. In recent news reports, we learn that “Apple thinks carefully about its entire product lineup” [30], that “Apple wants owners to sell their old iPhones back to the company for a discount on a new phone” [31], and that “Apple intends to work with record labels to identify and promote up and coming artists” [32]. In cases like these, people apply words normally associated with the psychological states of an individual person—words like ‘thinks’, ‘wants’, and ‘intends’—to a corporation as a whole. These same expressions can also be applied to other sorts of group agents. People talk about what a government agency ‘intends’, what a religious organization ‘thinks’, or what a sports team ‘loves’ or ‘hates’ [33]–[37]. Indeed, archival studies show that people speak about groups using mental state words spontaneously, even outside the context of an experiment [36], and cross-cultural studies document the use of mental state words in descriptions of groups not only in the West, but also in East Asian cultures [35], [37].

Does the use of such language indicate that people understand governments and other organizations by attributing mental states to a group? Critically, there are two different senses in which one might think about ‘groups’ and, accordingly, two different senses in which one might investigate the processes perceivers use to understand groups. On one hand, one could think about a ‘group’ as referring to the *members* of groups. If each group member is a human being, then the group is simply a collection of human beings. A first sense in which one might investigate how perceivers understand groups, then, is to investigate how people understand collections of human beings. On the other hand, one could think about a ‘group’ as referring to a *group agent* [38], [39]. A group agent itself is not merely a collection of separate human beings but, instead, an entity with whatever sort of status attaches itself to corporations, nations, and sports teams. Thus, a second sense in which one might investigate how perceivers understand groups is to investigate how people understand not collections of individuals, but group agents.

An example highlights the distinction between a group in the sense of a collection of individuals and a group in the sense of a group agent. Consider the sentence “The employees and stockholders of Acme Corp. are all in debt.” This sentence says something about the financial condition of various individual human beings while making no claims about the financial condition of the corporation with which they are associated. In other words, the sentence ascribes a property to the members without ascribing that property to the group agent itself. By contrast, consider the sentence, “Acme Corp. is in debt.” This sentence says something about the financial condition of a corporation, but it makes no claims at all about the financial condition of any individual human beings. (The corporation itself could be in debt even if all of the employees and stockholders were in excellent financial shape.) Thus, this sentence ascribes a property to a group agent without ascribing that same property to any of the members.

Existing work already provides some evidence for the claim thinking about groups in the first sense—i.e., thinking about collections of human beings—shares properties and processes with thinking about individual people. Behaviorally, the vast literatures on stereotypes and intergroup relations show that people are willing to ascribe psychological attributes to whole collections of others [7], [40]–[45], and studies indicate that some of the same principles that apply to the ascription of properties to individual agents also appear in the ascription of properties to whole collections of agents [46], [47]. Moreover, a recent neuroimaging study observed activation in brain regions associated with theory-of-mind—MPFC, TPJ, and precuneus—when participants evaluated the applicability of certain preferences both to individual people and to collections of individuals, compared to a non-mental control condition [48]. Taken together, these behavioral and neuroimaging studies provide support for the view that people can ascribe psychological attributes not only to individual human beings but also to collections of human beings, and that they may use similar processes to do so (even if the outcomes of those processes may sometimes differ [47], [49]).

Yet studies like these still leave open the question of how people understand groups in the second sense—i.e., how they understand group agents. As we saw above, people can ascribe a non-mental property to all of the members of a group agent without ascribing that property to the group agent itself (“All of the employees and stockholders are in debt”). Similarly, perhaps people can ascribe a mental property (i.e., a mental state) to all of the members of a group without in any way ascribing these states to the group agent itself (“The employees and stockholders all love Jeopardy!”). We have also seen that people can ascribe a non-mental property to a group without ascribing that property to the individual members (“Acme Corp. is in debt.”). Similarly, perhaps people can ascribe mental states to a group agent without ascribing that state to any of the members. Indeed, recent research suggests that the more people perceive a ‘group mind’, the less they tend to perceive the minds of the members of that group [8], [50].

With this in mind, the current studies investigate how perceivers understand group agents by examining the extent to which understanding group agents shares important properties and processes with understanding individuals. Experiment 1 examines behaviorally the extent to which people ascribe mental states to group agents over and above attributions of mental states to their individual members. Experiment 2 uses fMRI to investigate the extent to which understanding and predicting the behavior of group agents recruits brain regions associated with understanding and predicting the behavior of individuals—i.e., brain regions associated with theory of mind.

## Experiment 1: Ascriptions to group agents vs. ascriptions to group members

When people use sentences that appear to ascribe mental states to a group agent, are they actually ascribing something to the group agent, or are they merely attributing something to the group's members? For example, consider the sentence, “United Food Corp. believes that the new policy is morally unacceptable.” At least on the surface, this sentence appears to attribute a mental state (the belief that the policy is morally unacceptable) to a group agent (United Food Corp). However, it is possible that this is just a linguistic shortcut, and that when people use or hear sentences like this one, they are really attributing mental states to the members of the group, not to the group itself.

Existing research demonstrates that people sometimes do use sentences that appear to attribute a property to a group when referring to its members, specifically when the members of the group have the particular property in their roles as group members [39]. For example, if each member of the Sigma Chi fraternity gets drunk, and if each of them does so in his role as a Sigma Chi member, people tend to agree with the sentence, “The Sigma Chi fraternity got drunk” [39]. This sentence appears on the surface to be ascribing a property to the fraternity itself—the actual organization— but is in fact just a shorthand way of ascribing a property to the individual members in their roles as members.

In Experiment 1, we examine whether apparent mental state attributions to group agents can involve attributions of a property to a group agent itself, or whether they reduce to attributions to individual group members. To the extent that perceivers genuinely attribute a property to the group agent itself, attributions to group agents should sometimes diverge from attributions to the members of those groups. That is, we should observe (a) cases in which perceivers attribute a mental state to all of the members of the group without attributing that state to the group agent itself and (b) cases in which perceivers attribute a mental state to the group agent without attributing that state to any of the group's members. In contrast, to the extent that apparent attributions to group agents are merely shorthand for attributions to the group members, participants should not attribute properties to the group agent that they do not also attribute to the members of the group. Thus, finding that individuals attribute mental states to a group agent without attributing that state to any of the group's members would be the most unambiguous evidence that perceivers can apply mental states to group agents themselves.

### Method

#### Participants

116 Yale students and faculty (33% female; age range 18-54, mean age 21 years) were recruited outside a dining hall to fill out a questionnaire for payment.

#### Ethics statement

This study was approved by the Institutional Review Board at Yale University. All participants provided written informed consent.

#### Materials and Procedure

This experiment used a 2 (mental state: individual-only or group-only) × 3 (question: any member, each member, group) design in which target was manipulated within-subject and question type was manipulated between subjects. Each participant received eight vignettes in counterbalanced order. Four vignettes were designed in such a way that it would be logically possible to ascribe a particular mental state to each of the individuals in the group without ascribing that state to the group itself (*Individual-only* condition). For example, one vignette described an organization devoted to fighting the death penalty. All of the members of this anti-death penalty organization are also interested in antebellum American history, so they decide to form a separate organization, with exactly the same members, called the Shady Grove Antebellum Historical Society (SGAHS), which meets to discuss historical questions. If participants are willing to ascribe a mental state to all of the individual members without ascribing that mental state to the group as a whole, participants should report that all of the members of SGAHS want to fight the death penalty but that the SGAHS itself *does not* want to fight the death penalty. On the other hand, to the extent that attributions to a group simply reduce to the attributions made to the individual members, participants should report that SGAHS *does* want to fight the death penalty.

The other four vignettes were designed such that that it would be logically possible to ascribe a mental state to the group itself without ascribing that state to any of the individual members (*Group-only* condition). For example, one vignette described a large organization that was commissioned to build a space shuttle. Some members of the organization put together the software, others build the exterior, still others are in charge of the fuel, and so forth. But there is no single person who works on every aspect of the project. To the extent that people are willing to ascribe a property to a group agent over and above its members, participants should say that the organization knows how to build a space shuttle, but the individual members do not. In another vignette, a Community Association needs to choose music for an upcoming event. Some members really want to play punk music and can't stand classical, others really want to play classical music but strongly dislike punk, so in the end, the Association selects a third option: classic rock. If people are willing to attribute properties to group agents over and above their members, participants should say that the Community Association itself preferred playing classic rock but that none of the individual members shared this preference. On the other hand, to the extent that attributions to the group simply reduce to the attributions made to the individual members, participants should report either that most or all of the individual members prefer playing classic rock or that the group itself does not prefer playing classic rock. For full texts of the vignettes, see (Text S1).

Each participant was randomly assigned to one of three question conditions: ‘any member,’ ‘each member,’ or ‘group.’ Participants in the ‘any member’ condition received after each vignette a question about whether any individual member of the group had a particular mental state (‘Do any of the members of the Community Association prefer the idea of playing classic rock to the idea of playing every other type of music?’). Participants in the ‘each member’ condition were asked whether *each* member had the relevant state (‘Do each of the individual members of the Community Association prefer…?’). Finally, participants in the ‘group’ condition received questions about whether the group itself had the relevant state (‘Does the Community Association prefer…?’). Each question was answered on a scale from 1 (‘No’) to 7 (‘Yes’).

### Results

**Figure 1:**
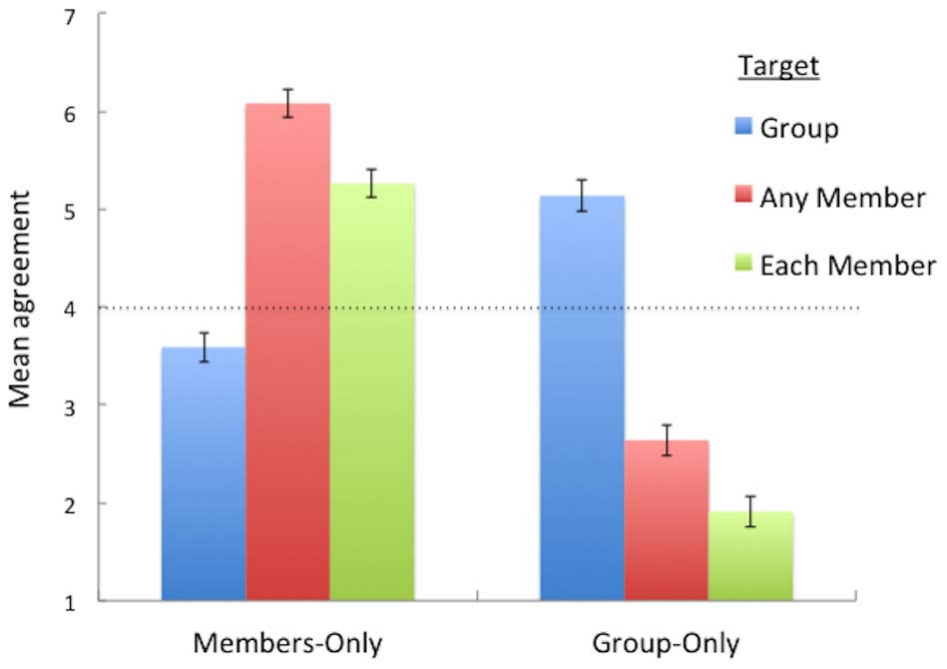
Mean agreement with mental state ascriptions by condition for the Members-Only and Group-Only vignettes. Error bars show SE mean. Dotted black line indicates neutral midpoint; points above indicate agreement and points below indicate disagreement.

Two participants failed to complete all items of the questionnaire. We calculated the mean response to ‘group’, ‘any member’, and ‘each member’ questions in the ‘Members Only’ vignettes and the ‘Group Only’ vignettes for the remaining participants (see Fig. 1). To the extent that participants attributed purported mental states to group agents themselves, we should observe both cases in which participants attribute a state to all of the members of the group without attributing that state to the group itself and, most critically, cases in which participants attribute a state to the group itself without attributing that state to any of the individual members. See (Table S1) for complete dataset.

For the Members-Only vignettes, a one-way ANOVA revealed a significant effect of question condition, *F*(2, 114)  =  41.2, *p* < .001, η^2^ =  .42 (Fig. 1), such that participants were willing to attribute states to some or all of the members of a group without attributing those states to the group itself. Tukey's posthoc tests showed that participants agreed less with ascriptions in the ‘group’ question condition than in either the ‘any member’ question condition, *p* < .001, or the ‘each member’ question condition, *p* < .001, suggesting that attributions to the group did not simply reduce to attributions to the group's members.

Critically, for the Group-Only vignettes, a one-way ANOVA again revealed a significant effect of question condition on participants' responses, *F*(2, 114)  =  91.6, *p* < .001, η^2^ =  .62 (Fig. 1), such that participants were willing to attribute states to the group itself that they did not attribute to any of the members of the group. Tukey's posthoc tests showed that participants agreed more with ascriptions in the ‘group’ question condition than in either the ‘any member’ question condition, *p* < .001, or the ‘each member’ question condition, *p* < .001. Moreover, participants' responses in the group question condition were significantly above the neutral midpoint of the scale, *p* < .001, indicating that participants were genuinely endorsing sentences ascribing mental states to group agents. These results suggest that attributions to the group agent were made over and above the attributions made to individual members.

This study explored the relationship between ascribing states to group agents and their members. We observed cases in which participants attributed a state to all of the members but did not attribute that state to the group itself and also cases in which participants attributed a state to the group itself but did not attribute the state to any of the members. Together, these results demonstrate that mental state ascriptions to a group agent can diverge from those made to the group's individual members, suggesting that perceivers can attribute a property of some sort to the group agent itself.

## Experiment 2: Neural processes supporting mental state ascriptions to group agents

Experiment 1 suggests that that when people use expressions of the form ‘United Food Corp. wants.’, they appear to be ascribing something to the group itself, rather than to the members of the group. However, a further question concerns the processes supporting these ascriptions. That is, although such statements clearly involve the same linguistic expressions that people use when applying theory-of-mind to individual human beings, to what extent do they also involve the same cognitive processes?

To investigate the processes supporting attributions of purported mental states to group agents, we scanned participants using fMRI as they considered the mental states of individuals and groups. In one task, participants read sentences that referred explicitly to the mental states of groups and individuals (along with matched, non-mental control sentences). In a second task, participants carried out a procedure that relied on mental state ascription incidentally, without the use of mental state words: making predictions about what an individual or group would do in a variety of situations. To the extent that perceivers rely on processes associated with understanding individuals when they understand and predict the behavior of groups, brain regions associated with theory-of-mind should be active both when thinking about individuals and when thinking about group agents, and they should be active to a similar degree. On the other hand, to the extent that perceivers rely on different processes to understand group agents, we should observe reduced activation in brain regions associated with theory-of-mind—RTPJ, MPFC, and precuneus—during consideration of groups versus individuals. In the design of this study, steps were taken to (a) minimize, as much as possible, the likelihood that participants would simply consider the minds of individual group members when considering group agents and (b) test sensitively the degree to which brain regions associated with theory of mind are engaged during consideration of group agents. Unlike past studies, no individuals were mentioned or shown in the group condition, and both directed and spontaneous theory of mind tasks were included. Moreover, the results of Experiment 1 show that perceivers do interpret sentences about group mental states as ascribing mental states to the group agent itself.

Although MPFC, TPJ, and precuneus have all been associated consistently with theory-of-mind, finer-grained differences in the response profiles of these regions facilitate predictions about their involvement during consideration of group agents. Recent neuroimaging research has increasingly revealed that, even when mental state attributions to individuals are concerned, MPFC, TPJ, and precuneus do not all respond in the same ways under the same circumstances. In particular, there are at least two ways in which the processes associated with purported mental state reasoning about group agents may differ from those associated with individual people. One is that certain properties of the *type* of mental state content being attributed may differ. The other is that certain properties of the *target* to whom that content is being attributed may differ.

The RTPJ consistently demonstrates sensitivity to the *type* of mental state being ascribed. Specifically, a series of studies has demonstrated that RTPJ is selective for processing representational mental states, such as beliefs [51]–[55]; see [56] for review. The RTPJ response is high when participants read stories that describe a character's true or false beliefs but low during stories containing other socially salient information, such as a character's physical appearance, cultural background, or even internal sensations such as hunger or fatigue [25]. Similarly, activation in RTPJ is higher during inferences about an individual's beliefs than during closely matched inferences about an individual's preferences regardless of whether such inferences are more or less constrained by external information—a response profile that is not shared by other regions associated with social cognition, such as MPFC [57]. Moreover, activation in the RTPJ consistently tracks with thinking about mental contents, not merely seeing mental state words. RTPJ becomes engaged when participants think about others' mental states even in the absence of any mental state words, such as when they view non-verbal cartoons [58] or read descriptions of actions that imply a particular mental state [22]. Conversely, mental state words alone do not elicit activation in the RTPJ; for review see [59]. Thus, mental state words are neither necessary nor sufficient for eliciting RTPJ activation. Instead, RTPJ activation during social cognition appears to be associated with the ascription of representational mental state content; for discussion see [60]–[62]. Thus, to the extent that perceivers attribute representational mental states to group agents, we should observe similar levels of RTPJ activation during consideration of group agents and individuals, both of which should exceed that associated with a non-mental control condition.

In contrast, MPFC appears to be especially sensitive to the *target* of mental state ascription. In particular, thinking about oneself, a similar individual, a familiar individual, or an individual whose perspective one has taken earlier is associated with more MPFC activation than thinking about more distant others [63]–[67]. MPFC also appears to be sensitive to the target of consideration when theory-of-mind is not explicitly called for. For example, this region exhibits less activation during consideration of “dehumanized” than “humanized” individuals [68] and responds more during consideration of one's own versus another person's physical attributes [26]. Although it remains open to further inquiry whether lower MPFC response in these cases genuinely indexes a difference in the degree to which mental states are attributed [68] or rather the use of an alternative process for doing so [57], [63], [67], the sensitivity of MPFC to the target of judgment suggests that group agents may be particularly likely to be associated with lower activation than individuals in this region.

### Method

#### Participants

Nineteen right-handed, native English speakers (10 female; age range 19-25, mean age 21 years) with no history of neurological problems participated for payment. All participants had normal or corrected-to-normal vision.

#### Ethics statement

This study was approved by the Committee on the Use of Humans as Experimental Subjects (COUHES) at the Massachusetts Institute of Technology. All participants provided written informed consent.

#### Stimuli and Behavioral Procedure

##### Directed theory-of-mind task

During fMRI scanning, participants completed an individual vs. group agent theory-of-mind task in which they read short statements about everyday events. Participants were instructed to read each statement and were told that they would be asked a series of questions about the statements later on in the experiment. Inanimate (control) statements communicated information without reference to people (e.g., “Although there wasn't much real data on agricultural production, the statistics showed that rutabaga production was consistently going down.”). Based on each control statement, an *individual* statement and a *group* statement were constructed. *Individual* statements concerned a single person's mental state (e.g., “Although there wasn't much real data on agricultural production, George Hailwood was sure that rutabaga production was going down.”). *Group* statements concerned the ‘mental state’ of a group agent (e.g., “Although there wasn't much real data on agricultural production, United Food Corp. was sure that rutabaga production was going down.”). No participant viewed more than one version of the same base statement.

In each run of this task, participants read statements organized around a single theme (e.g., one run concerned George Hailwood, United Food Corp., and food production, whereas another concerned Stephanie Ann Majors, a record company, and music sales). For full texts of the stimuli, see (Text S2). Participants completed ten functional runs of eighteen statements each (six per condition), totaling 180 trials. Statements were displayed in random order within each run and remained onscreen for 8 s. Trials were separated by a variable inter-stimulus interval (2–16 s) during which participants passively viewed a black screen.

##### Spontaneous theory-of-mind task

Following each run of the directed theory-of-mind task, participants were asked to make a series of predictions about the individual and group about which they had just read (e.g., “The asparagus might be contaminated by bacteria. Would George Hailwood [United Food Corp.] be more likely to (a) recall all of the asparagus or (b) cover up the whole incident?”). This task elicited mental state reasoning indirectly by asking participants to formulate predictions about behavior, such that no mental state words were presented to participants at any point. Each question remained onscreen for 12 s, and participants were obliged to respond during that time by pressing one of two buttons on a button box held in the left hand. Each run comprised eight trials (four per condition) separated by 10 s. Each participant answered each question either for the individual or the group, but not both (question assignment randomized across participants).

##### Theory-of-mind localizer

In order to facilitate region-of-interest (ROI) analyses focusing on brain regions associated with theory-of-mind, participants also completed a functional localizer task in which they read short narratives and made inferences about individual protagonists' beliefs (e.g., concerning the location of a hidden object) and inferences about physical representations (e.g., the contents of an outdated photograph [22]). Each narrative was displayed for 10 s and was followed by a statement that participants judged as true or false (e.g., Belief story: “Sarah thinks her shoes are under the dress”; Physical story: “The original photograph shows the apple on the ground”) which remained onscreen for 4 s. Participants were obliged to respond during that time by pressing one of two buttons. Trials were separated by 12 s fixation. Participants completed four runs, each of which comprised eight trials (four per condition), for a total of 32 trials.

#### Imaging Procedure

fMRI data were collected using a 3 Tesla Siemens scanner. Functional imaging used a gradient-echo echo-planar pulse sequence (TR  =  2 s; TE  =  30 ms; flip angle  =  90°, 30 near-axial slices, 4 mm thick, in-plane resolution  =  3×3 mm, whole brain coverage). These sequences used PACE online motion correction for movement < 8 mm. fMRI data were preprocessed and analyzed using SPM2 (Wellcome Department of Cognitive Neurology, London, United Kingdom) and custom software. Data from each subject were motion corrected and normalized into a standard anatomical space based on the ICBM 152 brain template (Montreal Neurological Institute). Normalized data were then spatially smoothed (5 mm full-width-at-half-maximum [FWHM]) using a Gaussian kernel.

Statistical analyses were performed using the general linear model in which the event-related design was modeled using a canonical hemodynamic response function and other covariates of no interest (a session mean and a linear trend). After these analyses were performed individually for each participant, the resulting contrast images for each participant (i.e., *individual > control, group > control*) were entered into a second-level analysis in which participants were treated as a random effect. Data were thresholded at *p*<.001, *k*>10, uncorrected.

For the directed theory of mind task, conjunction analysis was performed following the procedure described by Cabeza, Dolcos, Graham, & Nyberg [69]. Whole-brain statistical maps were created from the *individual* > *control* and *group* > *control* contrasts separately to identify voxels activated by each condition (thresholded individually at *p* < .01), making for a conjoint threshold of *p* < .001.

ROIs were defined for each subject individually based on a whole-brain analysis of the theory-of-mind localizer in three regions: RTPJ, precuneus, and MPFC. Regions were defined as 10 or more contiguous voxels that were significantly more active (*p* < 0.001, uncorrected) during stories about mental states than during control stories about physical representations. The average responses relative to rest during the individual and group conditions were then estimated in these regions. Within each ROI, the mean percent signal change (PSC  = 100 × raw BOLD magnitude for [condition − rest]/raw BOLD magnitude for rest) was calculated for each condition at each time point (averaging across all voxels in the ROI and all trials of the same condition) and averaged across seconds 6–10 to account for hemodynamic lag. Individual subject means for each condition of each task are available as (Table S2). The full fMRI dataset is available upon request.

### Results

#### Directed theory-of-mind task

In order to assess the extent to which common cognitive processes subserve thinking about the minds of individuals and groups, we first conducted whole-brain, random effects analyses of BOLD signal. In whole-brain analyses, activation when participants contemplated the mental states of both individuals and groups (compared to control) was observed in brain regions associated with theory-of-mind, including MPFC, RTPJ, and precuneus. The direct comparisons between the individual and group conditions (individual <> group) yielded no areas of differential activation in regions typically associated with social cognition (Table 1). To the extent that overlapping BOLD activation reflects the engagement of overlapping cognitive processes, these initial observations suggest that thinking about individuals and groups may draw upon shared theory-of-mind processes.

**Table 1:** Regions emerging from whole brain analyses.

Region	*x*	*y*	*z*	*T* value
***Theory-of-mind Localizer (Belief > Photo)***				
PC	2	−64	42	10.73
Right TPJ	58	−54	34	6.38
MPFC	0	52	46	6.27
Right STS	56	−26	−10	5.74
Left TPJ	−48	−52	20	5.39
Left Anterior STS	−54	4	−24	5.00
Left STS	−54	−20	−14	4.64
Right Temporal Pole	54	6	−34	4.51
Left Temporal Pole	−36	16	−26	4.39
***Individual > Control***				
PC	−6	−68	38	8.73
Right TPJ	48	−58	34	6.66
Left Middle Frontal Gyrus	−30	54	4	6.22
Left Inferior Parietal Lobule	−40	−66	42	6.04
Right Middle Frontal Gyrus	56	20	36	4.20
Orbitofrontal cortex	4	50	−18	4.27*
MPFC	−2	52	40	4.13*
Left Middle Temporal Gyrus	−60	−30	−10	3.97
***Group > Control***				
PC	2	−62	36	7.76
Right TPJ	54	−64	32	5.75
Right Temporal Pole	46	16	−32	5.71
MPFC	−6	54	42	4.85
Right Middle Frontal Gyrus	44	24	28	4.65
Left Inferior Parietal Lobule	−44	−66	42	4.44
MPFC	−10	42	50	4.27
***Individual + Group > Control***				
PC	0	−60	36	8.45
Right TPJ	48	−58	32	6.32
Left Inferior Parietal Lobule	−42	−66	42	5.60
Left Middle Frontal Gyrus	−32	54	6	5.17
Right Middle Frontal Gyrus	44	24	28	4.94
MPFC	−6	56	44	4.73
***Individual > Group***				
Right Middle Frontal Gyrus	36	54	6	5.25
Right Posterior Middle Frontal Gyrus	26	12	50	4.87
Left Inferior Parietal Lobule	−46	−56	58	4.32
***Group > Individual***				
Right Middle Occipital Gyrus	44	−80	14	4.81
Right Fusiform Gyrus	36	−74	−14	4.69
Left Middle Frontal Gyrus	−52	4	40	4.25
Left Posterior Middle Temporal Gyrus	−54	−56	2	4.04

Average peak voxels for regions identified in whole-brain random effects analysis (*p* < .001, *k* > 10 voxels; * =  *p* < .005, k > 10 voxels) of the localizer and directed individual vs. group theory-of-mind task in Montreal Neurological Institute (MNI) coordinates. TPJ  =  temporal parietal junction; PC  =  precuneus; MPFC  =  medial prefrontal cortex; STS  =  superior temporal sulcus.

**Figure 2:**
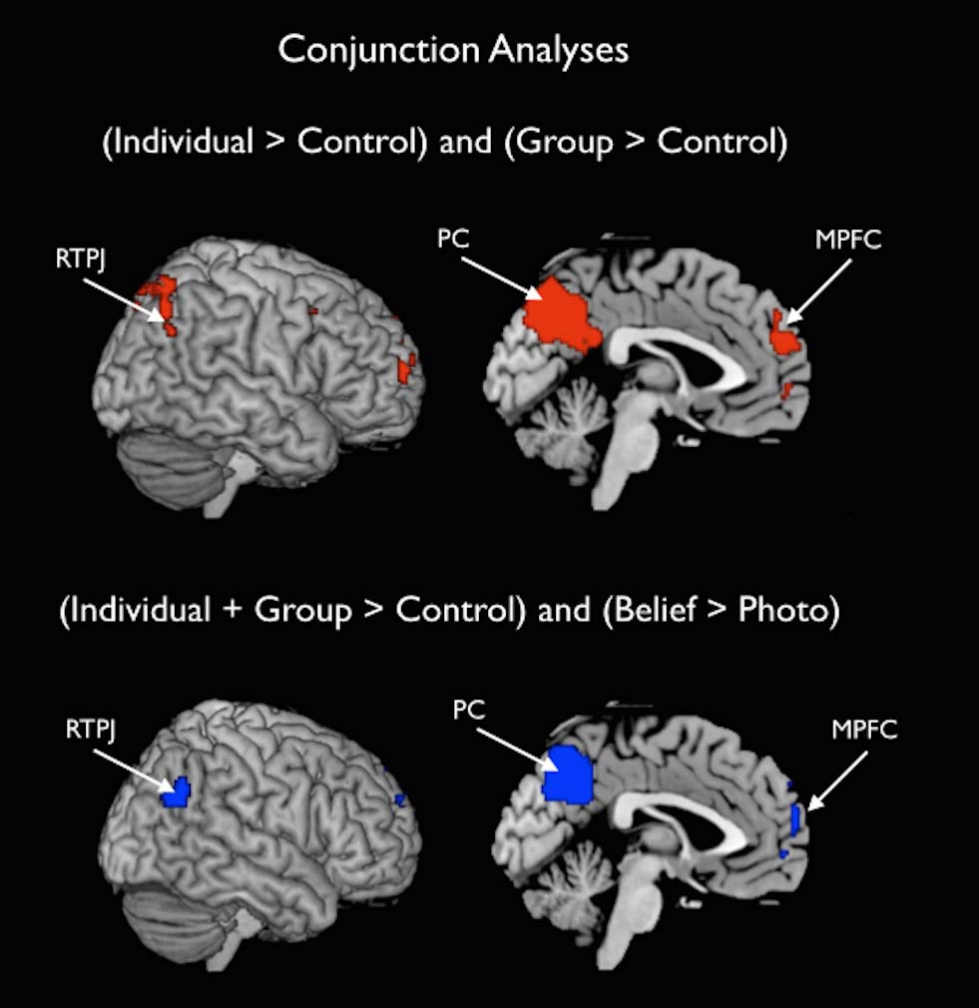
Conjunction analyses. Top: A conjunction analysis revealed conjoint activation in MPFC, TPJ (bilaterally), and precuneus when participants read about the mental states of individuals and groups, compared to a non-mental control condition. Bottom: These regions also overlapped with those recruited by the theory-of-mind localizer. Activations are displayed on a canonical brain image.

Next, to test more directly the extent to which overlapping regions of cortex were recruited during contemplation of the mental states of individuals and groups, we conducted a conjunction analysis on the individual > control and group > control contrasts. This analysis revealed conjoint activation specifically in brain regions associated with theory-of-mind–MPFC, right and left TPJ, and precuneus–suggesting further that thinking about individuals and groups draw upon shared processes (Table 2; Fig. 2).

**Table 2:** Regions emerging from the conjunction analysis.

Region	X	y	Z
PC	0	−60	36
Right TPJ	48	−60	32
Right Middle Frontal Gyrus	44	24	28
Right Middle Frontal Gyrus	52	16	46
MPFC	12	56	10
MPFC	−6	54	42
Left Middle Frontal Gyrus	−28	52	10
Left Middle Frontal Gyrus	−38	54	−2
Left Middle Frontal Gyrus	−52	20	38
Left Anterior Superior Temporal Gyrus	−34	6	−24
Left TPJ	−52	−66	28
Left Inferior Parietal Lobule	−42	−66	42
Left Middle Temporal Gyrus	−60	−28	−10

Average peak voxels for regions identified in whole-brain conjunction analysis of the individual > control and group > control contrasts (*p* < .01 for each) in Montreal Neurological Institute (MNI) coordinates. TPJ  =  temporal parietal junction; PC  =  precuneus; MPFC  =  medial prefrontal cortex.

**Figure 3:**
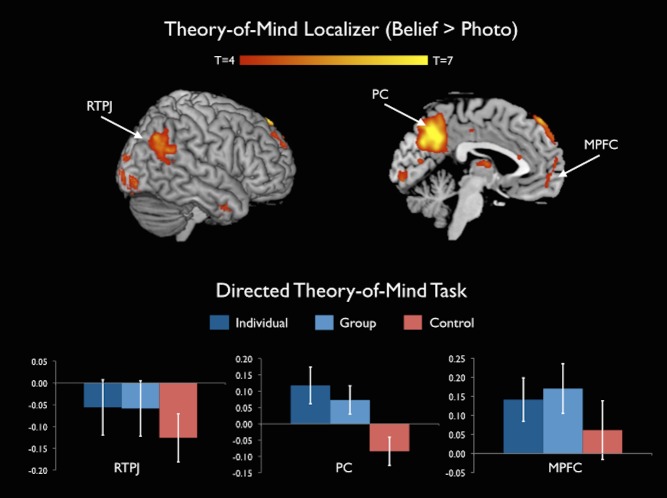
Regions identified by the theory-of-mind localizer. Top: Brain regions emerging from the theory-of-mind localizer (belief > photo; *p* < .001, uncorrected, *k* > 10). Activations are displayed on a canonical brain image. Bottom: Percent signal change (PSC) in BOLD response during the individual, group, and control conditions of the directed theory-of-mind task in regions identified by the independent theory-of-mind localizer.

Although the foregoing analyses suggest that similar processes subserve thinking about individuals and groups as compared to a control condition, they leave open the possibility that thinking about individual and group agents may recruit theory-of-mind processes to different degrees. In order to evaluate the degree to which processes associated with theory-of-mind were recruited when thinking about individuals versus groups, we conducted independent region-of-interest (ROI) analyses within the regions of MPFC, RTPJ, and precuneus identified by the independent theory-of-mind localizer. Because the mental states in the localizer task were attributed to individual protagonists, this analysis technique provides a particularly stringent test for whether thinking about group agents genuinely recruits processes associated with thinking about individuals. Consistent with previous research, the theory-of-mind localizer (belief > photo contrast) yielded activation in MPFC (17/19 participants), RTPJ (19/19 participants), and precuneus (19/19 participants); Fig. 3. First, ROI analyses of the main task confirmed that each of these regions showed greater activation in the individual condition than in the control condition (MPFC, *t*(16) = 2.28, *p* < .04, *d*  =  0.57; Right TPJ, *t*(18) = 2.43, *p* < .03, *d*  =  0.57; precuneus, *t*(18) = 5.99, *p* < .0001, *d*  =  1.41). Second, ROI analyses further revealed that each of these regions showed greater activation in the group condition as compared to control (MPFC, *t*(16) = 2.22, *p* < .04, *d*  =  0.55; Right TPJ, *t*(18) = 2.39, *p* < .03, *d*  =  0.56; precuneus, *t*(18) = 6.32, *p* < .0001, *d*  =  1.49). Finally, no significant differences were observed between the responses to individuals versus groups in any of these regions, (MPFC, *t*(16) = 0.69, *p*  =  .5; Right TPJ, *t*(18) = 0.09, *p*  =  .93; precuneus, *t*(18) =  1.51, *p*  =  .15; Fig. 3). Together, these analyses suggest that brain regions associated with theory-of-mind are recruited to a highly similar degree during the contemplation of individuals and groups.

#### Spontaneous theory-of-mind task

The design of the previous task raises the possibility that activation during the individual and group conditions may have differed from the control condition due to the explicit use of mental state words (e.g., thinks, believes, wants) in the individual and group conditions. To explore whether common theory-of-mind processes subserve attributions to individuals and groups even when no mental state terms are used, we analyzed data from the portion of the study during which participants made predictions about the behavior of individuals and groups. Specifically, we compared activation during the individual and group conditions of the prediction task in the same regions of RTPJ, MPFC, and precuneus identified by the theory-of-mind localizer. Results replicated those from the directed theory-of-mind task. Consistent with the hypothesis that thinking about the minds of individuals and groups recruit similar theory-of-mind processes, activations above baseline were observed across the network in both the individual, *t*(19)  =  2.84, *p* < .02, *d*  =  0.65, and the group condition, *t*(19)  =  2.23, *p* < .04, *d*  =  0.51 (averaging across regions), and no differences were observed between the individual and group conditions in RTPJ (*M*_ind_  =  −.004 *M*_group_  =  −.019, *t*(19)  =  0.86, *p* > .39), MPFC (*M*_ind_  =  .197 *M*_group_  =  .180, *t*(19)  =  0.36, *p* > .72), or precuneus (*M*_ind_  =  .266 *M*_group_  =  .231, *t*(19)  =  1.64, *p* > .12). For individual subject data, see (Table S2). These results suggest that the similar patterns of activation in the individual and group conditions observed in the first task are not simply due to the common use of mental state terms in those conditions. Here, when no mental state terms were presented, making predictions about individual and group agents' behavior also recruited the theory-of-mind network to an indistinguishable degree.

### Discussion

In describing corporations, government agencies and other organizations, people sometimes use sentences of the form ‘Apple thinks…’ or ‘The CIA wants…’ The aim of the present investigation was to help illuminate how people think about group agents. The results of Experiment 1 indicate that sentences like these are ascribing something to the group agent itself. Perceivers used expressions like ‘believes’ and ‘wants,’ not merely to talk about some or all of the individual members of a group, but to talk about the group agent. Thus, attributions to the group sometimes diverged from attributions to the individual members: participants were willing to attribute a state to the group itself even when they were not willing to attribute that state to any of the individual members, and they were willing to attribute a mental state to all members of a group even when they were not willing to attribute that state to the group itself. In turn, the results of Experiment 2 reveal that that such ascriptions recruit brain regions associated with thinking about the minds of individuals, i.e., brain regions associated with theory-of-mind, both when theory-of-mind use is called for explicitly and when it arises spontaneously.

Past research has demonstrated consistent engagement of a particular network of regions, including MPFC, RTPJ, and precuneus, during inferences about the minds of individual people, i.e., during theory-of-mind. Across two tasks, we observed activation in this network when participants read or made predictions about group agents. In the *directed theory-of-mind task*, participants read about the states of individuals, group agents, and inanimate objects. In the *spontaneous theory-of-mind* task, participants made predictions about what individual or group agents would do in particular situations. In both cases, activation associated with groups was indistinguishable from that associated with consideration of individuals. Whole-brain analyses, conjunction analysis, and ROI analyses all support the conclusion that cognitive processes associated with thinking about the minds of individuals were also recruited when participants thought about the ‘mind’ of a group agent. However, it is worth noting the possibility that participants may have been thinking to some degree about the minds of individual group members, and that this may have accounted for the observed activation in theory-of-mind regions during consideration of group agents. This possibility is weakened, but not completely ruled out, by (a) the fact that, unlike past studies, no individuals were mentioned or shown in the group condition and (b) the observation that perceivers interpret sentences about group mental states as ascribing mental states to the group agent itself in Experiment 1, and (c) the recent observation that the more perceivers think about the ‘mind’ of the group, the less they think about the minds of its members [8].

Past research has documented the selectivity of the RTPJ for attributing representational mental content, such as beliefs and intentions, to others [22], [25], [57], [61], [62], compared to other sorts of attributions, such as those concerning a person's physical appearance, preferences, or personality traits. In this research, neither the mere presence of a person nor the need to make other types of inferences about that person was associated with as much activation in this region as attributing representational mental states. Accordingly, the fact that the RTPJ activated indistinguishably during consideration of individuals and groups (but distinguished both from the inanimate control condition) is an especially compelling suggestion that participants used similar processes for understanding the representational mental states of individuals and group agents.

Although the specific contributions of MPFC to social cognition remain uncertain, this region has been observed to be sensitive to the *target* of mental state ascription. In particular, greater MPFC activation has been associated with interpersonally close others [63]–[67], and with humanized others [68], compared to those who are more distant or dehumanized. Accordingly, it would not have been surprising to observe reduced MPFC response to group agents compared to individuals. However, the current study observed indistinguishable engagement during consideration of group agents and individuals in a region of MPFC involved in attributing mental states to individuals, as identified by the theory-of-mind localizer, and similar to regions of MPFC associated with mentalizing or theory-of-mind in past studies (according to Neurosynth [70]). Moreover, the individual condition and group condition were associated with greater MPFC activation than the inanimate control condition, suggesting that MPFC's contributions to individual-oriented social cognition are also present during social cognition concerning group agents.

More generally, an abundance of past research has observed greater engagement of brain regions associated with theory-of-mind when perceivers think about certain types of target entities (humans and, to some degree, other animals) than when they think about other types of target entities (computers, food, furniture); for reviews, see [71]–[73]. Here, we find just as much activation in brain regions associated with theory-of-mind when people think about group agents as when they think about individual humans, yet a group agent is something very different from a human being or animal, or even from a collection of human beings. Accordingly, the current results are consistent with the possibility that perceivers apply theory-of-mind generally to things that conform to a certain kind of abstract structure [13], [74], and that group agents turn out to be among the things that conform to that structure [75]. This possibility draws further support from recent research observing activation in brain regions associated with theory-of-mind during consideration of other non-human agents that display human-like properties [76]–[78] and is broadly consistent with the observation that brain regions engaged when people construct representations of others' mental states are also engaged when people construct other types of representations that are removed from their current, first-person experience, such as representations of the past or future [79]–[82].

In sum, people appear in certain respects to treat groups as ‘entities’ [47]. They assign moral blame to whole organizations as a whole [1], treat whole financial markets as though they have minds of their own [83], and give corporations many of the legal rights enjoyed by individual human beings [4]. In the current studies, we observed that perceivers were willing to attribute mental states to group agents that they did not attribute to the individual members of those groups, and that attributing mental states to group agents was associated with activation in the same brain regions that support ascriptions of mental states to individual people (as confirmed by an independent localizer task). Taken together, these results suggest that in order to understand the striking ways in which people reason about corporations, governments, and other group agents, it may be important to consider the possibility that perceivers sometimes attribute mental states such as beliefs, desires, and intentions not only to the members of such groups but also to the group agent itself.

## Supporting Information

Text S1 **Stimuli from Experiment 1.** Full text of all vignettes and questions.
(PDF)

Text S2 **Stimuli from Experiment 2.** Full text of the statements and questions from the directed and spontaneous theory-of-mind tasks.
(PDF)

Table S1 **Data from Experiment 1.** Individual subject responses for each vignette. Condition 1 = ‘any member’; 2 = ‘each member’; 3 = ‘group’.
(PDF)

Table S2 **Data from Experiment 2.** Mean percent signal change (PSC) for each subject in each condition of the directed and spontaneous theory-of-mind tasks in regions identified by the theory-of-mind localizer.
(PDF)
