# Supplementary material for: The Neural Bases of Directed and Spontaneous Mental State Attributions to Group Agents
Source: PLoS One. 2014 Aug 20;9(8):e105341. doi: 10.1371/journal.pone.0105341 (PMC4139375; doi:10.1371/journal.pone.0105341)
Supplement: Text S1 — Stimuli from Experiment 1. Full text of all vignettes and questions. (PDF) [file pone.0105341.s001.pdf]

Experiment 1 Stimuli

Members-Only Vignettes

The National Association for the Promotion of Children's Music (NAPCM) is a non-profit organization that helps bring music to disadvantaged children. Members of the organization are sent to inner city schools to help lead children in sing-alongs of hits like 'Old McDonald Had a Farm' and 'London Bridge is Falling Down.'

As it happens, though, each of the members of NAPCM started out as an experimental noise musician and only ended up getting a day job at NAPCM when it became clear that it would not be possible to make a living in experimental noise rock. They each sit at home after work listening again and again to noise rock classics and playing ear-splitting melodies on guitar.

The NAPCM members have never mentioned this musical interest to each other. When they are at work, they focus only on implementing NAPCM's mission: 'To bring cheerful melodies to those who are less fortunate.'

- Does any individual member of NAPCM like experimental noise rock?
- Does NAPCM like experimental noise rock?
- Does each individual member of NAPCM like experimental noise rock?

Imagine a band named Dissonance Reduction. It has a singer, a drummer and a guitarist.

The singer is going through a nasty divorce, the drummer just lost a lot of money on the stock market, and the guitarist has a serious case of clinical depression. Each of them sometime go home after practice and start sobbing uncontrollably.

However, Dissonance Reduction itself has been extraordinarily successful. It has lots of new fans, and it might soon get signed to a good label.

- Are any of the members of Dissonance Reduction unhappy?
- Is the band Dissonance Reduction unhappy?
- Are each of the individual members of band Dissonance Reduction unhappy?

The National Cribbage Association (NCA) is in charge of monitoring the annual cribbage tournament. The NCA has consistently done an impressive job of making sure that all the games proceed fairly.

As it happens, though, all of the members of the NCA this year are from Connecticut. As they sit in their various homes watching videos of the latest cribbage bouts, they sometimes shout 'Go Connecticut!' And when a player from Connecticut wins an important match, they sometimes call up their friends and family to share the good news.

But the NCA itself has never taken any steps that might benefit players from Connecticut. In fact, the members of the NCA have never even told each other which states they are from.

- Does any individual member of the NCA want Connecticut players to win?
- Does the NCA want Connecticut players to win?
- Does each individual member of the NCA want Connecticut players to win?

A group of citizens banded together and formed an organization called Shady Grove Citizens Against the Death Penalty (SGCADP). Every Thursday night, SGCADP would convene to work on behalf of people who had been convicted of capital crimes.

But then one day, it became apparent that all of the members of SGCADP also avidly studied history. So they formed a new organization called the Shady Grove Antebellum Historical Society (SGAHS).

SGAHS had exactly the same members as SGCADP, but it did not engage in any political activities. It just met on Tuesday nights to promote interest in Shady Grove's historical traditions.

- Did any individual member of SGAHS want to fight the death penalty?
- Did SGAHS want to fight the death penalty?
- Did each individual member of SGAHS want to fight the death penalty?

Group-Only Vignettes

Imagine that the Community Association has been put in charge of the music for an upcoming event. The decision is made by a vote of the Community Association membership.

Some of the members always listen to hardcore punk music and would never play classical music. Other members always play classical music and would never go near hardcore punk.

So, in the end, the Community Association arrives at a compromise. It releases an official decision that the only music to be played at the event will be classic rock.

- Do any of the members of the Community Association prefer the idea of playing classic rock to the idea of playing every other type of music?
- Does the Community Association prefer the idea of playing classic rock to the idea of playing every other type of music?
- Do each of the individual members of the Community Association prefer the idea of playing classic rock to the idea of playing every other type of music?

The Cactus County Death Mongers was a drug running gang. The Death Mongers had three different people assigned to deal cocaine in the projects – Steve, Abigail and Adam.

At one point, it became clear that the Death Mongers had too many people assigned to cocaine distribution. So a meeting was called. Each gang member could vote for one of the three cocaine dealers. Then, whoever received the lowest number of votes would be booted out of the gang.

On the day of the meeting, people began calling out names: 'I vote for Adam.' 'I vote for Abigail.' 'I vote for Adam as well.' When all of the votes were tallied, it was clear that Steve had received the lowest number. He immediately left the room and no longer had any contact with the gang.

- Did any individual member of the Death Mongers decide to boot Steve out of the gang?
- Did the Death Mongers decide to boot Steve out of the gang?
- Did each individual member of the Death Mongers decide to boot Steve out of the gang?

Sometimes the Refreshments Committee of the Unity Baptist Church faces a difficult question. According to the bylaws, the Refreshments Committee must then follow the average of the suggestions of the individual members.

For example, the committee was once faced with a question about how many bagels to order. One member said: 'I suggest twenty-five.' Another said: 'I suggest twenty-six.' And the last one said: 'I suggest thirty.'

Each of the members stood by his or her specific suggestion. However, the committee staff promptly went out and ordered twenty-seven bagels.

- Did any individual member of the committee choose to order twenty-seven bagels?
- Did the committee choose to order twenty-seven bagels?
- Did each individual member of the committee choose to order twenty-seven bagels?

Imagine a vast organization that has been commissioned to build a space shuttle.

Some members of the organization are in charge of putting together the software, others are in charge of building the exterior, still others are in charge of the fuel, and so forth. But there is no single person who works on every aspect of the project.

The organization ends up doing an excellent job, and the space shuttle launch goes exactly as planned.

- Does any member of the organization know how to build a space shuttle?
- Does the organization know how to build a space shuttle?
- Does each individual member of the organization know how to build a space shuttle?
