# Supplementary material for: The Neural Bases of Directed and Spontaneous Mental State Attributions to Group Agents
Source: PLoS One. 2014 Aug 20;9(8):e105341. doi: 10.1371/journal.pone.0105341 (PMC4139375; doi:10.1371/journal.pone.0105341)
Supplement: Text S2 — Stimuli from Experiment 2. Full text of the statements and questions from the directed and spontaneous theory-of-mind tasks. (PDF) [file pone.0105341.s002.pdf]

Set 1: United Food Corp.

Directed Theory-of-Mind Task

1a. [Individual] Some apple orchards in the area had been running into insect problems, but George Hailwood was sure that he could eliminate these difficulties using pesticides.

1b. [Group] Some apple orchards in the area had been running into insect problems, but United Food Corp. was sure that it could eliminate these difficulties using pesticides.

1c. [Control] Some apple orchards in the area had been running into insect problems, but these difficulties were eliminated using pesticides.

2a. [Individual] The stock market had been doing well for so many years. Although there were already a few signs of trouble, George Hailwood thought that stocks would continue to go up.

2b. [Group] The stock market had been doing well for so many years. Although there were already a few signs of trouble, United Food Corp. thought that stocks would continue to go up.

2c. [Control] The stock market had been doing well for so many years, and there were complex spreadsheets filled with precise information about the value of each stock.

3a. [Individual] There was an outbreak of E. coli in the spinach. But George Hailwood truly expects that no one will be harmed if this spinach goes to market.

3b. [Group] There was an outbreak of E. coli in the spinach. But United Food Corp. truly expects that no one will be harmed if this spinach goes to market.

3c. [Control] There was an outbreak of E. coli in the spinach. But the available research reports suggest that no one was harmed when this spinach went to market.

4a. [Individual] In any given year, one crop always ended up doing the best. This time, George Hailwood thought it would probably be the potatoes.

4b. [Group] In any given year, one crop always ended up doing the best. This time, United Food Corp. thought it would probably be the potatoes.

4c. [Control] In any given year, one crop always ended up doing the best. This year, it ended up being the potatoes.

5a. [Individual] The information about the new ruling was supposed to be kept secret, but George Hailwood already knew exactly what it would say.

5b. [Group] The information about the new ruling was supposed to be kept secret, but United Food Corp. already knew exactly what it would say.

5c. [Control] The information about the new ruling was supposed to be kept secret, and it was therefore stored on a password-protected computer.

6a. [Individual] The new plan was a risky and dangerous one, and George Hailwood was genuinely uncertain whether it would work or not.

6b. [Group] The new plan was a risky and dangerous one, and United Food Corp. was genuinely uncertain whether it would work or not.

6c. [Control] The new plan was a risky and dangerous one, and even the complex mathematical models could not determine whether it would work or not.

7a. [Individual] Some had suggested that asparagus would never sell in today's market, but George Hailwood thought it really might be possible to make a killing in asparagus sales.

7b. [Group] Some had suggested that asparagus would never sell in today's market, but United Food Corp. thought it really might be possible to make a killing in asparagus sales.

7c. [Control] It is difficult to sell asparagus in today's market, but the use of highly successful marketing techniques has allowed some to make a killing in asparagus sales.

8a. [Individual] Although there wasn't much real data on agricultural production, George Hailwood was completely sure that rutabaga production was going down.

8b. [Group] Although there wasn't much real data on agricultural production, United Food Corp. was completely sure that rutabaga production was going down.

8c. [Control] Although there wasn't much real data on agricultural production, the statistics showed that rutabaga production was consistently going down.

9a. [Individual] It was time to adopt a new marketing plan. George Hailwood thought it might be a good idea to get leading hip hop artists to endorse his brussels sprouts.

9b. [Group] It was time to adopt a new marketing plan. United Food Corp. thought it might be a good idea to get leading hip hop artists to endorse their brussels sprouts.

9c. [Control] It was time to adopt a new marketing plan. The computerized marketing plan generator suggested getting leading hip hop artists to endorse the brussels sprouts.

10a. [Individual] The corn had been planted in areas that sometimes suffered from droughts. So George Hailwood was very happy that there was enough rain this year.

10b. [Group] The corn had been planted in areas that sometimes suffered from droughts. So United Food Corp. was very happy that there was enough rain this year.

10c. [Control] The corn had been planted in areas that sometimes suffered from droughts. Fortunately, though, there was enough rain this year.

11a. [Individual] The new technology was efficient and inexpensive. Still, George Hailwood was displeased that it might prove harmful to the environment.

11b. [Group] The new technology was efficient and inexpensive. Still, United Food Corp. was displeased that it might prove harmful to the environment.

11c. [Control] The new technology was efficient and inexpensive. The one problem was that it ended up proving harmful to the environment.

12a. [Individual] A rival corporation had been buying up much of the usable land. George Hailwood was thrilled that this corporation was now in serious trouble.

12b. [Group] A rival corporation had been buying up much of the usable land. United Food Corp. was thrilled that this corporation was now in serious trouble.

12c. [Control] A rival corporation had been buying up much of the usable land. But then this rival corporation had some difficulties and is now in serious trouble.

13a. [Individual] A clerical mistake led to a serious problem on a nearby farm. George Hailwood was very disappointed by this error and the problems it caused.

13b. [Group] A clerical mistake led to a serious problem on a nearby farm. United Food Corp. was disappointed by this error and the problems it caused.

13c. [Control] A clerical mistake led to a serious problem on a nearby farm. Indeed, this error caused the farm to lose a lot of money.

14a. [Individual] The price of tomatoes had been fluctuating, but George Hailwood is excited that it has been staying consistently high throughout the year.

14b. [Group] The price of tomatoes had been fluctuating, but United Food Corp. is excited that it has been staying consistently high throughout the year.

14c. [Control] The price of tomatoes had been fluctuating, but then the market stabilized and the price stayed consistently high throughout the year.

15a. [Individual] The judge officially found for the prosecution. Needless to say, George Hailwood was dismayed by this ruling.

15b. [Group] The judge officially found for the prosecution. Needless to say, United Food Corp. was dismayed by this ruling.

15c. [Control] The judge officially found for the prosecution. Needless to say, the defendant lost a large sum of money.

16a. [Individual] All signs indicated that stock prices would continue to go up. So George Hailwood was shocked that they were actually going down.

16b. [Group] All signs indicated that stock prices would continue to go up. So United Food Corp. was shocked that they were actually going down.

16c. [Control] All signs indicated that stock prices would continue to go up, but in the bad economic climate that winter, they were actually going down.

17a. [Individual] When sales of wheat continued to fall, George Hailwood was a bit discouraged, and he therefore discontinued the program.

17b. [Group] When sales of wheat continued to fall, United Food Corp. was a bit discouraged, and it therefore discontinued the program.

17c. [Control] When sales of wheat continued to fall, the total amount of revenue got to be too low, and the program was discontinued.

18a. [Individual] Although people continue to buy vegetables, George Hailwood is disappointed that they don't spend nearly as much on vegetables as they did in the old days.

18b. [Group] Although people continue to buy vegetables, United Food Corp. is disappointed that they don't spend nearly as much on vegetables as they did in the old days.

18c. [Control] Although people continue to buy vegetables, a series of recent studies show that they don't spend nearly as much on vegetables as they did in the old days.

Spontaneous Theory-of-Mind Task

1. The asparagus might be contaminated by bacteria. Would United Food Corp. [George Hailwood] be more likely to (a) recall all the asparagus or (b) try to cover up the whole incident?

2. A new video shows Brittney Spears eating zucchini. Would United Food Corp. [George Hailwood] be more likely to (a) suddenly increase zucchini production or (b) keep production at the same levels?

3. The Canadians consistently outsell Americans in the cabbage market. Would United Food Corp. [George Hailwood] be more likely to (a) fight for protectionist tariffs or (b) improve the quality of its cabbage?

4. Some have suggested that one might be able to make cushions out of broccoli. Would United Food Corp. [George Hailwood] be more likely to (a) seriously explore this possibility or (b) reject it out of hand?

5. The latest harvest was lambasted in the trade press. Would United Food Corp. [George Hailwood] be more likely to (a) write a forceful letter to the editor or (b) just wait and let the whole issue pass?

6. The greatest cucumber expert of this century just passed away. Would United Food Corp. [George Hailwood] be more likely to (a) officially commemorate his death or (b) just carry on business as usual?

7. The latest episode of 'South Park' implies that celery is unhealthy. Would United Food Corp. [George Hailwood] be more likely to (a) put out a press release or (b) just send an email to its own employees?

8. It is time for a new banana advertisement. Would United Food Corp. [George Hailwood] be more likely to (a) use scenes of pirates eating bananas or (b) just explain the various health benefits?

Set 2: Decorations Committee

Directed Theory-of-Mind Task

1a. [Individual] The curtains still had not arrived by Wednesday, but Mary Beth Simmons was completely certain they would be there by Friday.

1b. [Group] The curtains still had not arrived by Wednesday, but the Decorations Committee was completely certain they would be there by Friday.

1c. [Control] The curtains still had not arrived by Wednesday, but the official legal contract said that they would have to be there by Friday.

2a. [Individual] The atrium was a bit barren and austere, and Mary Beth Simmons thought it would be helpful to add a rug or something.

2b. [Group] The atrium was a bit barren and austere, and the Decorations Committee thought it would be helpful to add a rug or something.

2c. [Control] The atrium was a bit barren and austere, but it started to fill up nicely with the addition of the lamp, the bookcase, and the new rug.

3a. [Individual] When the economic downturn began, the budget was slashed, but Mary Beth Simmons was sure she would be able to muddle through somehow.

3b. [Group] When the economic downturn began, the budget was slashed, but the Decorations Committee was sure it would be able to muddle through somehow.

3c. [Control] When the economic downturn began, the budget was slashed, but it turned out that the proposed project was able to keep muddling through somehow.

4a [Individual] Although the policy would doubtless have been quite popular and successful, Mary Beth Simmons felt that it was unacceptable from a moral standpoint.

4b. [Group] Although the policy would doubtless have been quite popular and successful, the Decorations Committee felt that it was unacceptable from a moral standpoint.

4c. [Control] Although the policy would doubtless have been quite popular and successful, it was unacceptable from a moral standpoint and violated the organization's charter.

5a. [Individual] The whole decision ended up depending on what Mary Beth Simmons said. Unfortunately, Marilyn didn't even know that the issue had come up.

5b. [Group] The whole decision ended up depending on what the Decorations Committee said. Unfortunately, that subcommittee didn't even know that the issue had come up.
5c. [Control] The whole decision ended up depending on what the official contract said. Unfortunately, the contract included no provision for cases like the one at hand.

6a. [Individual] That vacuum cleaner had failed again and again. Yet Mary Beth Simmons felt sure that it would actually work correctly this one evening.

6b. [Group] That vacuum cleaner had failed again and again. Yet the Decorations Committee felt sure that it would actually work correctly this one evening.

6c. [Control] That vacuum cleaner had failed again and again. Yet if the filter was changed on time, it was sure to work correctly this one evening.

7a. [Individual] After the embezzlement charges were filed, the judge requested a report about where the money had gone. But Mary Beth Simmons truly did not know.

7b. [Group] After the embezzlement charges were filed, the judge requested a report about where the money had gone. But the Decorations Committee truly did not know.

7c. [Control] After the embezzlement charges were filed, the judge requested a report about where the money had gone. But the corporate spreadsheet did not have the answer.

8a. [Individual] Back in 1998, a few rats started getting into the kitchen. At the time, Mary Beth Simmons thought this was just a brief difficulty that would soon go away.

8b. [Group] Back in 1998, a few rats started getting into the kitchen. At the time, the Decorations Committee thought this was just a brief difficulty that would soon go away.

8c. [Control] Back in 1998, a few rats started getting into the kitchen. If the exterminator had been called, this would have been just a brief difficulty that went away soon.

9a. [Individual] The new wallpaper was covered with purple paisley shapes. Yet, oddly enough, Mary Beth Simmons was convinced that it looked good.

9b. [Group] The new wallpaper was covered with purple paisley shapes. Yet, oddly enough, the Decorations Committee was convinced that it looked good.

9c. [Control] The new wallpaper was covered with purple paisley shapes. Yet, oddly enough, it looked pretty good next to that old bookcase over by the fireplace.

10a. [Individual] The curtains that were supposed to be in the atrium ended up in the auditorium. Mary Beth Simmons deeply regrets this error.

10b. [Group] The curtains that were supposed to be in the atrium ended up in the auditorium. The Decorations Committee deeply regrets this error.

10c. [Control] The curtains that were supposed to be in the atrium ended up in the auditorium. But they will be moved over shortly.

11a. [Individual] The new wallpaper was just installed a few days ago. Mary Beth Simmons is pleased that it is very inexpensive.

11b. [Group] The new wallpaper was just installed a few days ago. The Decorations Committee is pleased to see that it is very inexpensive.

11c. [Control] The new wallpaper was just installed a few days ago. It is water-proof, durable and very inexpensive.

12a. [Individual] The paintings will not arrive for another three months. Mary Beth Simmons is dismayed that this is taking so long.

12b. [Group] The paintings will not arrive for another three months. The Decorations Committee is dismayed that this is taking so long.

12c. [Control] The paintings will not arrive for another three months. Only very rarely do the delivery arrangements take quite this long.

13a. [Individual] The price of flowers went up, then went back down. Mary Beth Simmons is happy that she will be able to get flowers at a good rate.

13b. [Group] The price of flowers went up, then went back down. The Decorations Committee is happy that it will be able to get flowers at a good rate.

13c. [Control] The price of flowers went up, then went back down. At this point, the going rate for tulips, roses, or chrysanthemums is actually quite good.

14a. [Individual] There are already six drapes, five curtains, and ten tapestries. Mary Beth Simmons is not in favor of buying anything further for that building.

14b. [Group] There are already six drapes, five curtains, and ten tapestries. The Decorations Committee is not in favor of buying anything further for that building.

14c. [Control] There are already six drapes, five curtains, and ten tapestries. So there is already far more than enough in the way of decoration in that building.

15a. [Individual] The new construction crew appeared to be highly professional. Mary Beth Simmons is shocked that they have been doing so badly.

15b. [Group] The new construction crew appeared to be highly professional. The Decorations Committee is shocked they have been doing so badly.

15c. [Control] The new construction crew appeared to be highly professional. Yet, despite its apparent promise, the crew has actually been doing very badly.

16a. [Individual] It appeared at first that the doorways with red trim would not be available. Mary Beth Simmons was thrilled that they are still in stock.

16b. [Group] It appeared at first that the doorways with red trim would not be available. The Decorations Committee was thrilled that they are still in stock.

16c. [Control] It appeared at first that the doorways with red trim would not be available. But because of fluctuations in annual sales, they are actually still in stock.

17a. [Individual] The new building looks magnificent on Autumn afternoons. So Mary Beth Simmons is disappointed that the building will be closed this Autumn.

17b. [Group] The new building looks magnificent on Autumn afternoons. So the Decorations Committee is disappointed that the building will be closed this Autumn.

17c. [Control] The new building looks magnificent on Autumn afternoons. But the truth is that, this year, the building will be closed for all of the Autumn months.

18a. [Individual] The chairs were built only ten years ago, and they are already starting to break. Mary Beth Simmons is unhappy with the quality of this product.

18b. [Group] The chairs were built only ten years ago, and they are already starting to break. The Decorations Committee is unhappy with the quality of this product.

18c. [Control] The chairs were built only ten years ago, and they are already starting to break. It would be advisable to buy a higher quality product.

Spontaneous Theory-of-Mind Task

1. Some cocaine was discovered underneath the rug. Would the Decorations Committee [Mary Beth Simmons] be more likely to (a) report it to the police or (b) sell it and keep the money?

2. There wasn't quite enough money available. Would the Decorations Committee [Mary Beth Simmons] be more likely to (a) decorate fewer rooms or (b) decorate each room less lavishly?

3. A used condom was discovered in the fireplace. Would the Decorations Committee [Mary Beth Simmons] be more likely to (a) ignore the whole issue or (b) ask someone how it got there?

4. The green drapes don't go with the purple rug. Would the Decorations Committee [Mary Beth Simmons] be more likely to (a) replace the drapes or (b) put in a gaudy chandelier to distract people?

5. The holiday party is coming up, and the guests need some dessert. Would the Decorations Committee [Mary Beth Simmons] be more likely to serve (a) chocolate eclairs or (b) raspberry tarts?

6. The office needs to be renovated. Would the Decorations Committee [Mary Beth Simmons] be more likely to select (a) wallpaper with pictures of trees or (b) wallpaper with pictures of elephants?

7. The desks need to be replaced. Would the Decorations Committee [Mary Beth Simmons] be more likely to select (a) a sleek, modern design or (b) a more old fashioned, institutional design?

8. A giant rat was found in the kitchen, right next to the refrigerator. Would the Decorations Committee [Mary Beth Simmons] be more likely to (a) tell the owners or (b) not tell anyone?

Set 3: Record Company

Directed Theory-of-Mind Task

1a. [Individual] The video was looking great, with some beautiful camerawork and editing. Stephanie Anne Majors thought it was sure to increase album sales.

1b. [Group] The video was looking great, with some beautiful camerawork and editing. The record label thought it was sure to increase album sales.

1c. [Control] The video was looking great, with some beautiful camerawork and editing. The marketing reports indicated that it would increase album sales.

2a. [Individual] The vocals had been recorded, but there were still some instrumentals missing. Stephanie Anne Majors recognized that the track needed a cello.

2b. [Group] The vocals had been recorded, but there were still some instrumentals missing. The record company recognized that the track needed a cello.

2c. [Group] The vocals had been recorded, but there were still some instrumentals missing. The track needed cello right after the end of the second verse.

3a. [Individual] Would the new album sell as well as the previous one? Stephanie Anne Majors was still unsure, but she thought sales would be at least acceptable.

3b. [Group] Would the new album sell as well as the previous one? The record company was still unsure, but it thought sales would be at least acceptable.

3c. [Control] Would the new album sell as well as the previous one? The marketing reports made no prediction, but they did say that sales would be at least acceptable.

4a. [Individual] That new band has no talent and no good songs. Yet, oddly enough, Stephanie Anne Majors continues to believe that they are destined for stardom.

4b. [Group] That new band has no talent and no good songs. Yet, oddly enough, the record company continues to believe that they are destined for stardom.

4c. [Control] That new band has no talent and no good songs. Yet oddly enough they continue to get all of these good gigs and are probably destined for stardom.

5a. [Individual] In the past, Stephanie Anne Majors has signed some acts that didn't do very well. But this time, she is completely certain that she has a winner on her hands.

5b. [Group] In the past, the record company has signed some acts that didn't do very well. But this time, the company is completely certain that it has a winner on its hands.

5c. [Control] In the past, the record company has signed some acts that didn't do very well. But this time, all of the sales figures indicate that it has a winner on its hands.

6a. [Individual] Different times call for different business practices. Right now, Stephanie Anne Majors honestly doesn't know what approach would be best.

6b. [Group] Different times call for different business practices. Right now, the record company honestly doesn’t know what approach would be best.

6c. [Control] Different times call for different business practices. Right now, things are changing fast, and the old practices are no longer effective.

7a. [Individual] The new computer system had been very expensive. Stephanie Anne Majors truly thought it would prove helpful, but it turned out to be a disaster.

7b. [Group] The new computer system had been very expensive. The record company truly thought it would prove helpful, but it turned out to be a disaster.

7c. [Control] The new computer system had been very expensive. An initial statistical analysis indicated that it would prove helpful, but it turned out to be a disaster.

8a. [Individual] It’s funny how quickly the music industry changes. Stephanie Anne Majors expected that she could keep her new policy in place, but it has to be replaced.

8b. [Group] It’s funny how quickly the music industry changes. The record company expected that it could keep it’s new policy in place, but it has to be replaced.

8c. [Control] It’s funny how quickly the music industry changes. That policy was only put into effect last year, but now it already has to be replaced.

9a. [Individual] Information seems to spread through the music world at an alarming rate. Stephanie Anne Majors already knows what that other band did yesterday.

9b. [Group] Information seems to spread through the music world at an alarming rate. The record company already knows what that other band did yesterday.

9c. [Control] Information seems to spread through the music world at an alarming rate. The newspaper is already reporting what that band did yesterday.

10a. [Individual] The new album had just been released and was now in stores. Stephanie Anne Majors was really happy that it was doing well.

10b. [Group] The new album had just been released and was now in stores. The record company was really happy that it was doing well.

10c. [Control] The new album had just been released and was now in stores. The recent sales report indicated that it was actually doing quite well.

11a. [Individual] The song had been entirely completed, with the exception of the violin solo. Stephanie Anne Majors was excited that the solo would be performed by a concert violinist.

11b. [Group] The song had been entirely completed, with the exception of the violin solo. The record company was excited that the solo would be performed by a concert violinist.

11c. [Control] The song had been entirely completed, with the exception of the violin solo. The official production plan specified that this solo would be performed by a concert violinist.

12a. [Individual] The new music video had just been completed, Stephanie Anne Majors was disappointed that it didn't really have the right look.

12b. [Group] The new music video had just been completed, but the record company was disappointed that it didn't really have the right look.

12c. [Control] The new music video had just been completed, but it didn't really have the right look, and it did not appear to be increasing album sales.

13a. [Individual] The album had not been selling very well, and Stephanie Anne Majors was upset that she was making so little money off of it.

13b. [Group] The album had not been selling very well, and the record company was upset that it was making so little money off of it.

13c. [Control] The album had not been selling very well, and the corporate sales reports revealed very little in the way of official revenue.

14a. [Individual] Everything was now going forward smoothly. Stephanie Anne Majors loved the new tracks and was happy that more were on their way.

14b. [Group] Everything was now going forward smoothly. The record company loved the new tracks and was happy that more were on their way.

14c. [Control] Everything was now going forward smoothly. The new tracks had just been completed, and more would soon be on their way.

15a. [Individual] The album just came out, but the title was misspelled on the front cover. Stephanie Anne Majors deeply regrets this error.

15b. [Group] The album just came out, but the title was misspelled on the front cover. The record company deeply regrets this error.

15c. [Control] The album just came out, but the title was misspelled on the front cover. It will be spelled correctly in the next edition.

16a. [Individual] The title track had just been recorded at the studio. Stephanie Anne Majors was displeased that the lead guitarist would not be appearing on it.

16b. [Group] The title track had just been recorded at the studio. The record company was displeased that the lead guitarist would not be appearing on it.

16c. [Control] The title track had just been recorded at the studio. Though the other songs all included drums and guitar, this one used only piano.

17a [Individual] Now that the production and mastering had been completed, the total cost of the album was available. Stephanie Anne Majors was shocked that it had cost so much.

17b. [Group] Now that the production and mastering had been completed, the total cost of the album was available. The record company was shocked that it had cost so much.

17c. [Control] Now that the production and mastering had been completed, the total cost of the album was available. The spreadsheet indicated that the cost had been quite high.

18a. [Individual] Now that the tour had begun, new t-shirts were being delivered. Stephanie Anne Majors was pleased that they were so artfully designed.

18b. [Group] Now that the tour had begun, new t-shirts were being delivered. The record company was pleased that they were so artfully designed.

18c. [Control] Now that the tour had begun, new t-shirts were being delivered. Unlike the ones from the last tour, this new batch was very artfully designed.

Spontaneous Theory-of-Mind Task

1. The latest hot new band does not sing at all but works entirely by lip synching. Would the record company [Stephanie Anne Majors] be more likely to (a) sign them immediately or (b) sign another band instead?

2. Profits from the reggae division have been declining for years. Would the record company [Stephanie Anne Majors] be more likely to (a) sell off the whole division or (b) continue operating but with reduced staff?

3. The latest band's t-shirts are crude and sexist. Would the record company [Stephanie Anne Majors] be more likely to (a) discontinue sales of them immediately or (b) wait until the first quarter's sales report was in?

4. The new album will be released on vinyl. Would the record company [Stephanie Anne Majors] be more likely to (a) create a lavishly illustrated artistic record sleeve or (b) just throw something together in a week?

5. One of the artists on the label was just caught dealing drugs. Would the record company [Stephanie Anne Majors] be more likely to (a) hire a publicist to hush things up or (b) simply terminate his or her contract?

6. The long term strategic plan has been completely failing. Would the record company [Stephanie Anne Majors] be more likely to (a) switch to a different approach or (b) lay off some of the employees?

7. The new gangsta rap album just went platinum. Would the record company [Stephanie Anne Majors] be more likely to (a) hold a big party for the employees or (b) just continue business as usual?

8. The seventeen year old pop star is wearing a very revealing outfit in her video. Would the record company [Stephanie Anne Majors] be more likely to (a) release it immediately or (b) first consult her parents?

Set 4: Math Department

Directed Theory-of-Mind Task

1a. [Individual] There were major cutbacks throughout the university, but Jeffrey Pettit thought that at least some programs would be spared.

1b. [Group] There were major cutbacks throughout the university, but the Department of Mathematics thought that at least some programs would be spared.

1c. [Control] There were major cutbacks throughout the university, but the computer spreadsheet indicated that at least some programs would be spared.

2a. [Individual] The new building was kind of cramped, and the plumbing was not working correctly. Still, Jeffrey Pettit thought it could easily be improved.

2b. [Group] The new building was kind of cramped, and the plumbing was not working correctly. Still, the Department of Mathematics thought it could easily be improved.

2c. [Control] The new building was kind of cramped, and the plumbing was not working correctly. Still, it seemed that the problems were small and could easily be fixed.

3a. [Individual] It seemed at first that the new kind of research was going nowhere, but Jeffrey Pettit was completely convinced that it is the wave of the future.

3b. [Group] It seemed at first that the new kind of research was going nowhere, but the Department of Mathematics was completely convinced that it is the wave of the future.

3c. [Control] It seemed at first that the new kind of research was going nowhere, but it is at least possible that it will really turn out to be the wave of the future.

4a. [Individual] This is a turbulent time in the study of algebraic topology. Jeffrey Pettit honestly wonders what the future will bring.

4b. [Group] This is a turbulent time in the study of algebraic topology. The Department of Mathematics honestly wonders what the future will bring.

4c. [Control] This is a turbulent time in the study of algebraic topology. Future research might move the field in any number of different ways.

5a. [Individual] The budget used to be pretty constant, but now Jeffrey Pettit truly doesn't know what is going to happen this coming year.

5b. [Group] The budget used to be pretty constant, but now the Department of Mathematics truly doesn't know what is going to happen this coming year.

5c. [Control] The budget used to be pretty constant, but now even the official departmental documents do not say anything about what is going to happen this coming year.

6a. [Individual] The planning office has now sent out its report. So Jeffrey Pettit actually knows exactly what will happen to the building.

6b. [Group] The planning office has now sent out its report. So the Department of Mathematics actually knows exactly what will happen to the building.

6c. [Control] The planning office has now sent out its report. So the Department of Mathematics has a document that says exactly what will happen to the building.

7a. [Individual] Financial information about students is supposed to be private, but Jeffrey Pettit actually knows a lot about how much money the students are making.

7b. [Group] Financial information about students is supposed to be private, but the Department of Mathematic actually knows a lot about how much money the students are making.

7c. [Control] Financial information about students is supposed to be private, but the documents describing how much money the students are making are actually on a public computer.

8a. [Individual] Funding for basic research had been decreasing for years, but Jeffrey Pettit was completely convinced that more money would somehow become available.

8b. [Group] Funding for basic research had been decreasing for years, but the Department of Mathematics was completely convinced that more money would somehow become available.

8c. [Control] Funding for basic research had been decreasing for years, but the department's computerized budgets continued to reflect the old financial information.

9a. [Individual] The university administration had rejected the request, but Jeffrey Pettit still believed he would find a way to make the new algebra program work.

9b. [Group] The university administration had rejected the request, but the Department of Mathematics still believed it would find a way to make the new algebra program work.

9c. [Control] The university administration had rejected the request, but the Department of Mathematics somehow found a way to make the new algebra program work.

10a. [Individual] The financial audit had just been completed. Jeffrey Pettit was deeply dismayed by the pattern of embezzlement in the records.

10b. [Group] The financial audit had just been completed. The Department of Mathematics was deeply dismayed by the pattern of embezzlement in the records.

10c. [Control] The financial audit had just been completed. Although the total revenue was fairly high, the finance reports clearly indicated a pattern of embezzlement.

11a. [Individual] A new proof was published in an important journal. Jeffrey Pettit was worried that it would end up overshadowing all the other work done that year.

11b. [Group] A new proof was published in an important journal. The Department of Mathematics was worried that it would end up overshadowing all the other work done that year.

11c. [Control] A new proof was published in an important journal. Because it was so original and unexpected, it ended up overshadowing all the other work done that year.

12a. [Individual] A number of students were admitted into the PhD program by mistake. Jeffrey Pettit truly regrets the inconvenience this caused.

12b. [Group] A number of students were admitted into the PhD program by mistake. The Department of Mathematics truly regrets the inconvenience this caused.

12c. [Control] A number of students were admitted into the PhD program by mistake. The error was due to a problem with the departmental computers.

13a. [Individual] A math professor at another university was denied tenure. Jeffrey Pettit was delighted to hear that she might now be available.

13b. [Group] A math professor at another university was denied tenure. The Department of Mathematics was delighted to hear that she might now be available.

13c. [Control] A math professor at another university was denied tenure. She is now available for hire and will probably be moving sometime soon.

14a. [Individual] The staff did not have very good training but ended up doing excellent work. Jeffrey Pettit is really impressed with their recent accomplishments.

14b. [Group] The staff did not have very good training but ended up doing excellent work. The Department of Mathematics is really impressed with their recent accomplishments.

14c. [Control] The staff did not have very good training but ended up doing excellent work. They actually went beyond the goals specified in the official planning document.

15a. [Individual] A great mathematician in China recently passed away. Jeffrey Pettit was deeply saddened by his departure.

15b. [Group] A great mathematician in China recently passed away. The Department of Mathematics was deeply saddened by his departure.

15c. [Control] A great mathematician in China recently passed away. The field of mathematics will never be quite the same after his departure.

16a. [Individual] A pet turtle was recently donated by one of the alumni. Jeffrey Pettit was, of course, delighted by the new gift.

16b. [Group] A pet turtle was recently donated by one of the alumni. The Department of Mathematics was, of course, delighted by the new gift.

16c. [Control] A pet turtle was recently donated by one of the alumni. The newsletter then included an appreciative mention of the gift.

17a. [Individual] A giant earthquake destroyed the building. Jeffrey Pettit was shocked and dismayed by the extent of the damage.

17b. [Group] A giant earthquake destroyed the building. The Department of Mathematics was shocked and dismayed by the extent of the damage.

17c. [Control] A giant earthquake destroyed the building. The damage was surprisingly extensive, leaving little of the building untouched.

18a. [Individual] A wave of scandals rocked the university community. Oddly enough, though, Jeffrey Pettit seemed strangely pleased by the whole affair.

18b. [Group] A wave of scandals rocked the university community. Oddly enough, though, the Department of Mathematics seemed strangely pleased by the whole affair.

18c. [Control] A wave of scandals rocked the university community. Yet no information could be found in the university’s official status reports, which focused entirely on other matters.

Spontaneous Theory-of-Mind Task

1. Budget problems are looming. Would the Department of Mathematics [Jeffrey Pettit] be more likely to (a) beg the university administration for more money or (b) lay off some of the administrative staff?

2. It might be possible to get more money by doing mathematical modeling for Iraq war. Would the Department of Mathematics [Jeffrey Pettit] be more likely to (a) take the contract or (b) refuse?

3. The government of North Korea wants access to the latest research. Would the Mathematics Department be more likely to (a) send them some papers or (b) keep the research private?

4. The mathematicians are moving into new offices. Would the Department of Mathematics [Jeffrey Pettit] be more likely to focus on making the offices (a) convenient or (b) aesthetically pleasing?

5. The whole university will probably go bankrupt. Would the Department of Mathematics [Jeffrey Pettit] be more likely to (a) do anything possible to avert disaster or (b) just stop working at all?

6. People in the finance department seem to be involved in corruption. Would the Department of Mathematics [Jeffrey Pettit] be more likely to (a) expose their crimes or (b) just cover everything up?

7. The most important conjecture of our time was just proven. Would the Department of Mathematics [Jeffrey Pettit] be more likely to (a) hold a celebration or (b) just continue work as usual?

8. A woman is applying for a position as a professor. Would the Department of Mathematics [Jeffrey Pettit] be more likely to judge her (a) entirely on merit or (b) in part based on her appearance?

Set 5: Swedish Army

Directed Theory-of-Mind Task

1a. [Individual] Despite the repeated accusations of child molestation among the ranks, Ingmar Andersson remains confident that his troops are entirely law-abiding.

1b. [Group] Despite the repeated accusations of child molestation among the ranks, the Swedish Army remains confident that its troops are entirely law-abiding.

1c. [Control] Despite the repeated accusations of child molestation among the ranks, the annual report continued to assert that the troops were entirely law-abiding.

2a. [Individual] At the moment, the only available food is that giant vat full of fish. Nonetheless, Ingmar Andersson is certain that something more appetizing will be available soon.

2b. [Group] At the moment, the only available food is that giant vat full of fish. Nonetheless, the Swedish Army is certain that something more appetizing will be available soon.

2c. [Control] At the moment, the only available food is that giant vat full of fish. Nonetheless, the inventory spreadsheet indicates that something more appetizing will be available soon.

3a. [Individual] The big competition is coming up soon, and everyone will be watching on TV. Ingmar Andersson is sure that he will be able to win.

3b. [Group] The big competition is coming up soon, and everyone will be watching on TV. The Swedish Army is sure that it will be able to win.

3c. [Control] The big competition is coming up soon, and everyone will be watching on TV. As it happens, the Swedes are very likely to win this year.

4a. [Individual] With the economy in shambles, everything is descending into chaos. Still, Ingmar Andersson is sure that he will be able to make it through.

4b. [Group] With the economy in shambles, everything is descending into chaos. Still, the Swedish Army is sure that it will be able to make it through.

4c. [Control] With the economy in shambles, everything is descending into chaos. Still, all evidence suggests that the Swedish Army will be able to make it through.

5a. [Individual] Troops had begun massing at the border, and there were already tanks on the Eastern front. Still, Ingmar Andersson genuinely expected that nothing would happen.

5b. [Group] Troops had begun massing at the border, and there were already tanks on the Eastern front. Still, the Swedish Army genuinely expected that nothing would happen.

5c. [Control] Troops had begun massing at the border, and there were already tanks on the Eastern front. But then, in the end, nothing actually happened.

6a. [Individual] The nation of Russia was becoming ever more powerful and aggressive. Yet Ingmar Andersson still continued to think that the Danes were the real enemy.

6b. [Group] The nation of Russia was becoming ever more powerful and aggressive. Yet the Swedish Army still continued to think that the Danes were the real enemy.

6c. [Control] The nation of Russia was becoming ever more powerful and aggressive. Yet it was still clear that the Danes were the real enemy.

7a. [Individual] This old military base is now swarming with rats and full of safety problems, but Ingmar Andersson still thinks that it is a good place to send soldiers for the holidays.

7b. [Group] This old military base is now swarming with rats and full of safety problems, but the Swedish Army still thinks that it is a good place to send soldiers for the holidays.

7c. [Control] This old military base is now swarming with rats and full of safety problems, but soldiers are still frequently sent there as a place to spend the holidays.

8a. [Individual] The terrorists escaped immediately, and there has been no sign of them since. Ingmar Andersson thinks they are probably hiding in the hills of Norway.

8b. [Group] The terrorists escaped immediately, and there has been no sign of them since. The Swedish Army thinks they are probably hiding in the hills of Norway.

8c. [Control] The terrorists escaped immediately, and there has been no sign of them since. As it happens, they ran off rapidly and are now hiding in the hills of Norway.

9a. [Individual] Once again, the newspaper reports are filled with stories of utter incompetence, but Ingmar Andersson continues to think that he can solve this problem swiftly and efficiently.

9b. [Group] Once again, the newspaper reports are filled with stories of utter incompetence, but the Swedish Army continues to think that it can solve this problem swiftly and efficiently.

9c. [Control] Once again, the newspaper reports are filled with stories of utter incompetence, but in this one case, the Swedish Army actually solved the problem swiftly and efficiently.

10a. [Individual] Now that he has made so many mistakes and the newspapers are filled with scandalous stories, Ingmar Andersson obviously regrets what he did.

10b. [Group] Now that it has made so many mistakes and the newspapers are filled with scandalous stories, the Swedish Army obviously regrets what it did.

10c. [Control] Now that it has made so many mistakes and the newspapers are filled with scandalous stories, the Swedish Army is clearly in big trouble.

11a. [Individual] It appears that one of the subcontractors left the chemical weapons in a local bar. Ingmar Andersson is extremely disappointed with the subcontractor's performance.

11b. [Group] It appears that one of the subcontractors left the chemical weapons in a local bar. The Swedish Army is extremely disappointed with the subcontractor's performance.

11c. [Control] It appears that one of the subcontractors left the chemical weapons in a local bar. That subcontractor certainly won't be getting any more contracts soon.

12a. [Individual] Once again, Ingmar Andersson received an award for good accounting practices. Needless to say, Ingmar is delighted by this news.

12b. [Group] Once again, the Swedish Army received an award for good accounting practices. Needless to say, the army is delighted by this news.

12c. [Control] Once again, the Swedish Army received an award for good accounting practices. Needless to say, the army newspaper is heavily publicizing this news.

13a. [Individual] In that period, drinking lots of martinis was all the rage, and as Ingmar Andersson has emphasized, he very much regrets some of the actions he took.

13b. [Group] In that period, drinking lots of martinis was all the rage, and as the Swedish Army has emphasized, it very much regrets some of the actions it took.

13c. [Control] In that period, drinking lots of martinis was all the rage, and as the Swedish Army has emphasized, it has now taken steps to curb alcohol consumption.

14a. [Individual] That new advertisement was supposed to be hip and engaging. Ingmar Andersson is therefore shocked and displeased that it has not helped the enlistment statistics.

14b. [Group] That new advertisement was supposed to be hip and engaging. The Swedish Army is therefore shocked and displeased that it has not helped the enlistment statistics.

14c. [Control] That new advertisement was supposed to be hip and engaging. But as it happens, systematic studies show that it has not actually helped the enlistment statistics.

15a. [Individual] The public target shooting contest will take place on Friday. Ingmar Andersson is afraid that he might not be quite as good at marksmanship as the Belgians.

15b. [Group] The public target shooting contest will take place on Friday. The Swedish Army is afraid that it might not be quite as good at marksmanship as the Belgians.

15c. [Control] The public target shooting contest will take place on Friday. The statistics show that the Swedish Army is not quite as good at marksmanship as the Belgians.

16a. [Individual] The administration just finished allocating materials. Ingmar Andersson was shocked that his regiment had been given horses instead of cars.

16b. [Group] The administration just finished allocating materials. The Swedish Army was shocked that it had been given horses instead of cars.

16c. [Control] The administration just finished allocating materials. The budget document indicated that the Swedish Army had been given horses instead of cars.

17a. [Individual] Military personnel are often exposed to harsh living conditions. Yet Ingmar Andersson remains dismayed that he cannot get access to decent espresso.

17b. [Group] Military personnel are often exposed to harsh living conditions. Yet the Swedish Army remains dismayed that it cannot get access to decent espresso.

17c. [Control] Military personnel are often exposed to harsh living conditions. For example, the Swedish Army has consistently been unable to get access to decent espresso.

18a. [Individual] The military brass band has always been forbidden from playing Rolling Stones songs. Ingmar Andersson is delighted that the band only plays old 50's music.

18b. [Group] The military brass band has always been forbidden from playing Rolling Stones songs. The Swedish Army is delighted that the band only plays old 50's music.

18c. [Control] The military brass band has always been forbidden from playing Rolling Stones songs. The regulations manual specifically only permits the use of old 50's music.

Spontaneous Theory-of-Mind Task

1. The Swedish government wants to allow gays into the army. Would the Swedish Army [Ingmar Andersson] be more likely to (a) launch a formal protest or (b) immediately agree?

2. There is a new policy putting stricter limits on the army's vodka supply. Would the Swedish Army [Ingmar Andersson] be more likely to (a) strictly enforce the policy or (b) let all violations go unpunished?

3. The barracks are suffering from an outbreak of genital herpes. Would the Swedish Army [Ingmar Andersson] be more likely to (a) implement a sex education program or (b) take no action at all?

4. The new uniforms are now being designed. Would the Swedish Army [Ingmar Andersson] be more likely to (a) choose the most cost efficient approach or (b) choose a more aesthetically pleasing design?

5. The annual soccer game with Finland is coming up. Would the Swedish Army [Ingmar Andersson] be more likely to (a) send troops to cheer in the stands or (b) just continue purely military work?

6. Some of the troops have been horribly wounded. Would the Swedish Army [Ingmar Andersson] be more likely to (a) support them for life or (b) only help out for a certain number of years?

7. The X47D machine malfunctions once every 5,000 uses. Would the Swedish Army [Ingmar Andersson] be more likely to (a) recall all of the machines now in use or (b) simply allow them to remain in place?

8. The meatballs tend to be kind of overcooked and don't taste very good. Would the Swedish Army [Ingmar Andersson] be more likely to (a) find and use a new recipe or (b) fire the head cook?

Set 6: Girl Scouts of America

Directed Theory-of-Mind Task

1a. [Individual] The newspapers were filled with stories about Catholic priests and altar boys, but Martha Weeks Shane honestly believed that her own credibility would be unaffected.

1b. [Group] The newspapers were filled with stories about Catholic priests and altar boys, but the Girl Scouts of America honestly believed that their own credibility would be unaffected.

1c. [Control] The newspapers were filled with stories about Catholic priests and altar boys, but it seems clear that the credibility of the Girl Scouts of America was completely unaffected.

2a. [Individual] The requirements for receiving a Sewing Badge had just gone down. Martha Weeks Shane thought children today might not be able to achieve true sewing excellence.

2b. [Group] The requirements for receiving a Sewing Badge had just gone down. The Girl Scouts of America thought children today might not be able to achieve true sewing excellence.

2c. [Control] The requirements for receiving a Sewing Badge had just gone down. Studies had shown that children today might not be able to achieve true sewing excellence.

3a. [Individual] Global warming was leading to higher temperatures in the winters. Martha Weeks Shane thought it might now be possible for her to run camping trips in November.

3b. [Group] Global warming was leading to higher temperatures in the winters. The Girl Scouts of America thought it might now be possible for them to run camping trips in November.

3c. [Control] Global warming was leading to higher temperatures in the winters. The Girl Scouts of America were now easily able to run camping trips all the way up to November.

4a. [Individual] Children were sounding increasingly out of tune when singing 'Make New Friends.' Martha Weeks Shane thought that the rise of the Spice Girls might be to blame.

4b. [Group] Children were sounding increasingly out of tune when singing ‘Make New Friends.’ The Girl Scouts of America thought that the rise of the Spice Girls might be to blame.

4c. [Control] Children were sounding increasingly out of tune when singing ‘Make New Friends.’ A systematic statistical analysis had tied this problem to the rise of the Spice Girls.

5a. [Individual] The evidence indicates that many junior high school girls use drugs. Still, Martha Weeks Shane thinks that there are no drugs in the lodges under her supervision.

5b. [Group] The evidence indicates that many junior high school girls use drugs. Still, the Girl Scouts of America thinks that there are no drugs in the lodges under its supervision.

5c. [Control] The evidence indicates that many junior high school girls use drugs. However, there have never been any drugs found in the designated girl scout lodges.

6a. [Individual] The end-of-the-year bake sales begin next week. Martha Weeks Shane thinks the best option would be to make those new purple brownies with the pink caramel sauce.

6b. [Group] The end-of-the-year bake sales begin next week. The Girl Scouts of America thinks the best option would be to make those new purple brownies with the pink caramel sauce.

6c. [Control] The end-of-the-year bake sales begin next week. There are only two new baked goods for this year: the purple brownies and the pink caramel sauce.

7a. [Individual] The new promotional materials needed a good picture. Martha Weeks Shane assumes that the best photos are still the ones that were taken in the 70s.

7b. [Group] The new promotional materials needed a good picture. The Girl Scouts of America assume that the best photos are still the ones that were taken in the 70s.

7c. [Control] The new promotional materials needed a good picture. However, the folder in the department office only contained the photos that were taken in the 70s.

8a. [Individual] What an astounding feat of organization! Martha Weeks Shane knows the size and color of every single one of the uniforms she has distributed in the past year.

8b. [Group] What an astounding feat of organization! The Girl Scouts of America knows size and color of every single one of the uniforms it has distributed in the past year.

8c. [Control] What an astounding feat of organization! The spreadsheet now lists the size and color of every single one of the uniforms that has been distributed in the past year.

9a. [Individual] The official girl scout promise is a long-standing tradition, and Martha Weeks Shane firmly believes that it is important to leave in the part about ‘God and my country.’

9b. [Group] The official girl scout promise is a long-standing tradition, and the Girl Scouts of America firmly believe that it is important to leave in the part about ‘God and my country.’

9c. [Control] The official girl scout promise is a long-standing tradition, and from its very first inception, it has always included that second verse about ‘God and my country.’

10a. [Individual] The president adopted a new healthy foods initiative. Martha Weeks Shane was deeply worried that sales of her cookies might radically decrease.

10b. [Group] The president adopted a new healthy foods initiative. The Girl Scouts of America was deeply worried that sales of their cookies might radically decrease.

10c. [Control] The president adopted a new healthy foods initiative. One result of this new initiative was a decrease in sales of Girl Scouts of America cookies.

11a. [Individual] The annual charity drive had just begun. Martha Weeks Shane was excited about involving people in a new initiative to help little kittens.

11b. [Group] The annual charity drive had just begun. The Girl Scouts of America was excited about involving people in a new initiative to help little kittens.

11c. [Control] The annual charity drive had just begun. The official newsletter explained that this year’s project would involve helping little kittens.

12a. [Individual] The only way to settle the question was to consult the corporate bylaws. Martha Weeks Shane was disappointed that these bylaws did not permit gay people to be scout leaders.

12b. [Group] The only way to settle the question was to consult the corporate bylaws. The Girl Scouts of America was disappointed that these bylaws did not permit gay people to be scout leaders.

12c. [Control] The only way to settle the question was to consult the corporate bylaws. As it happened, these bylaws did not permit gay people to be scout leaders.

13a. [Individual] The new programs had just been unveiled, and Martha Weeks Shane was very excited about the possibility of offering an official Filmmaking Badge.

13b. [Group] The new programs had just been unveiled, and the Girl Scouts of America was very excited about the possibility of offering an official Filmmaking Badge.

13c. [Control] The new programs had just been unveiled, and the annual bulletin indicated that certain troops would now be offering an official Filmmaking Badge.

14a. [Individual] There will be a talent show at the annual meeting, and Martha Weeks Shane is very excited that this year’s show will include a skit performed by robots.

14b. [Group] There will be a talent show at the annual meeting, and the Girl Scouts of America are very excited that this year's show will include a skit performed by robots.

14c. [Control] There will be a talent show at the annual meeting, and the official program specifically says that this year's show will include a skit performed by robots.

15a. [Individual] Girl scouts used to roast marshmallows over campfires, but Martha Weeks Shane is concerned that this practice might result in dangerous marshmallow poisoning.

15b. [Group] Girl scouts used to roast marshmallows over campfires, but the Girl Scouts of America is concerned that this practice might result in dangerous marshmallow poisoning.

15c. [Control] Girl scouts used to roast marshmallows over campfires, but recent experimental studies raise the possibility that this practice might result in dangerous marshmallow poisoning.

16a. [Individual] Summertime is approaching, and the annual summer trip is about to begin. Martha Weeks Shane is excited to see that so many of last year's campers will be returning.

16b. [Group] Summertime is approaching, and the annual summer trip is about to begin. The Girl Scouts of America are excited to see that so many of last year's campers will be returning.

16c. [Control] Summertime is approaching, and the annual summer trip is about to begin. The database established by the camp shows that many of last year's campers will be returning.

17a. [Individual] The archery and riflery programs are already in place. However, Martha Weeks Shane very much regrets that she will not be able to offer boxing to the girls this year.

17b. [Group] The archery and riflery programs are already in place. However, the Girl Scouts of America very much regrets that it will not be able to offer boxing to the girls this year.

17c. [Control] The archery and riflery programs are already in place. However, the activities section of the bulletin shows that there will not be any programs available on boxing this year.

18a. [Individual] The new leadership has been doing a great job of organizing camping trips and other such activities. Still, Martha Weeks Shane is disappointed that revenue has not gone up.

18b. [Group] The new leadership has been doing a great job of organizing camping trips and other such activities. Still, the Girl Scouts of America is disappointed that revenue has not gone up.

18c. [Control] The new leadership has been doing a great job of organizing camping trips and other such activities. Still, the latest budget statistics show that revenue has not gone up.

Spontaneous Theory-of-Mind Task

1. The main spokesperson has recently become quite overweight. Would the Girl Scouts of America [Martha Weeks Shane] be more likely to (a) replace her or (b) keep her on in her present position?

2. The traditional girl scout songs are going out of style. Would the Girl Scouts of America [Martha Weeks Shane] be more likely to (a) put together some trendier songs or (b) just stick with the old standbys?

3. The executive director has just died of AIDS. Would the Girl Scouts of America [Martha Weeks Shane] be more likely to (a) release a moving and touching obituary or (b) just hush the whole thing up?

4. The girl scouts have just received a major grant. Would the Girl Scouts of America [Martha Weeks Shane] be more likely to (a) take some time to celebrate or (b) move forward immediately with new initiatives?

5. A major rock band has released a song making fun of girl scouts. Would the Girl Scouts of America [Martha Weeks Shane] be more likely to (a) denounce the song in a press release or (b) contact the band directly?

6. Girl scout cookies aren't selling as well as glossy photos. Would the Girl Scouts of America [Martha Weeks Shane] be more likely to (a) disband the whole cookie program or (b) just try to improve the recipes?

7. It's time to develop a new girl scout uniform. Would the Girl Scouts of America [Martha Weeks Shane] be more likely to (a) make the skirts substantially shorter or (b) add a colorful design?

8. The girl scouts face intense competition from church youth groups. Would the Girl Scouts of America [Martha Weeks Shane] be more likely to (a) adopt a more hip, trendy image or (b) hold events in evangelical churches?

Set 7: Boston Symphony Orchestra

Directed Theory-of-Mind Task

1a. [Individual] Competition for listeners was getting fierce. Jonathan Phillips thought the best approach might be to focus less on playing music and more on looking distinguished.

1b. [Group] Competition for listeners was getting fierce. The Symphony Orchestra thought the best approach might be to focus less on playing music and more on looking distinguished.

1c. [Control] Competition for listeners was getting fierce. Recent scientific studies indicated that the best approach was to focus less on playing music and more on looking distinguished.

2a. [Individual] The most well-attended concerts were always full of the same old boring stuff, but Jonathan Phillips knew that he ought to try something new this time.

2b. [Group] The most well-attended concerts were always full of the same old boring stuff, but the Symphony Orchestra knew that it ought to try something new this time.

2c. [Control] The most well-attended concerts were always full of the same old boring stuff, but the daily newspaper said that there was going to be something new this time.

3a. [Individual] Only twelve people had showed up for the Bulgarian composer series, but Jonathan Phillips expected that he could get better attendance for his Albanian themed fugues.

3b. [Group] Only twelve people had showed up for the Bulgarian composer series, but the Symphony Orchestra expected that it could get better attendance for its Albanian themed fugues.

3c. [Control] Only twelve people had showed up for the Bulgarian composer series, but marketing research showed that one might get better attendance for Albanian themed fugues.

4a. [Individual] Even sophisticated computer systems couldn’t tell the sound of those two violins apart, but Jonathan Phillips deemed that the older one sounded better.

4b. [Group] Even sophisticated computer systems couldn’t tell the sound of those two violins apart, but the Symphony Orchestra deemed that the older one sounded better.

4c. [Control] Even sophisticated computer systems couldn’t tell the sound of those two violins apart, but the sales report showed that older one was selling for a higher price.

5a. [Individual] Although the magazines usually ranked him in around fifth place overall, Jonathan Phillips thought that he was actually the second best in the nation.

5b. [Group] Although the magazines usually ranked it in around fifth place overall, the Symphony Orchestra thought that it was actually the second best in the nation.

5a. [Control] Although the magazines usually ranked it in around fifth place overall, the Symphony Orchestra was consistently second best in actual ticket sales.

6a. [Individual] Many of history's greatest musicians had been drug addicts. Yet Jonathan Phillips thought it would be best never to hire any addicts as musicians.

6b. [Group] Many of history's greatest musicians had been drug addicts. Yet the Symphony Orchestra thought it would be best never to hire any addicts as musicians.

6c. [Control] Many of history's greatest musicians had been drug addicts. Yet the bylaws specifically forbade hiring people engaged in that sort of activity as musicians.

7a. [Individual] Attendance at the concerts kept going down year after year, but Jonathan Phillips imagined that his work was getting better and better.

7b. [Group] Attendance at the concerts kept going down year after year, but the Symphony Orchestra imagined that its work was getting better and better.

7c. [Control] Attendance at the concerts kept going down year after year, but the truth is that the music had been continuing to get better and better.

8a. [Individual] Numerous pieces of classical music had drawn on the themes from literature. So Jonathan Phillips inferred that it might be good to turn next to theorems from mathematics.

8b. [Group] Numerous pieces of classical music had drawn on the themes from literature. So the Symphony Orchestra inferred that it might be good to turn next to theorems from mathematics.

8c. [Control] Numerous pieces of classical music had drawn on the themes from literature. But there had never been too much music drawing on theorems from mathematics.

9a. [Individual] Studies showed that fat people were better at singing Italian opera. Yet Jonathan Phillips firmly believed that fat people would be worse at singing Wagner.

9b. [Group] Studies showed that fat people were better at singing Italian opera. Yet the Symphony Orchestra firmly believed that fat people would be worse at singing Wagner.

9c. [Control] Studies showed that fat people were better at singing Italian opera. Yet more recent research was suggesting that fat people might actually be worse a singing Wagner.

10a. [Individual] There were occasionally calls to play some kind of 'non-traditional music.' However, Jonathan Phillips had always been displeased by orchestral versions of contemporary pop hits.

10b. [Group] There were occasionally calls to play some kind of 'non-traditional music.' However, the Symphony Orchestra had always been displeased by orchestral versions of contemporary pop hits.

10c. [Control] There were always calls to play some kind of ‘non-traditional music.’ However, the official bylaws said that the symphony could never play orchestral versions of contemporary pop hits.

11a. [Individual] The annual bassoon competition was coming up. Jonathan Phillips was delighted that he would have the chance to crush and destroy his rivals once again.

11b. [Group] The annual bassoon competition was coming up. The Symphony Orchestra was delighted that it would have the chance to crush and destroy its rivals once again.

11c. [Control] The annual bassoon competition was coming up. Thus far, the Symphony Orchestra had always succeeded in crushing and destroying all of its rivals at these events.

12a. [Individual] An outside auditing firm was now looking closely at the books. Jonathan Phillips worried that the irregularities in the entertainment budget would be detected.

12b. [Group] An outside auditing firm was now looking closely at the books. The Symphony Orchestra worried that the irregularities in the entertainment budget would be detected.

12c. [Control] An outside auditing firm was now looking closely at the books. Unfortunately, there had always been some very serious irregularities in the entertainment budget.

13a. [Individual] Ticket sales this year had been extraordinarily high, but Jonathan Phillips was disappointed that the concerts would never be as packed as they were in Wagner’s day.

13b. [Group] Ticket sales this year had been extraordinarily high, but the Symphony Orchestra was disappointed that the concerts would never be as packed as they were in Wagner’s day.

13c. [Control] Ticket sales this year had been extraordinarily high, but, of course, even the best concerts in modern times could never be as packed as they were in Wagner’s day.

14a. [Individual] The concerts had always been quite traditional, but Jonathan Phillips was excited that he could now do a ‘politically correct’ version of one of the old operas.

14b. [Group] The concerts had always been quite traditional, but the Symphony Orchestra was excited that he could now do a ‘politically correct’ version of one of the old operas.

14c. [Control] The concerts had always been quite traditional, but the latest schedule included a notice about the arrival of a new ‘politically correct’ version of one of the old operas.

15a. [Individual] The chairs out in the audience are extremely sturdy. Nonetheless, Jonathan Phillips worries that the audience members will get so fat that the seats will break.

15b. [Group] The chairs out in the audience are extremely sturdy. Nonetheless, the Symphony Orchestra worries that the audience members will get so fat that the seats will break.

15c. [Control] The chairs out in the audience are extremely sturdy. It's true that the audience members keep getting fatter and fatter, but those chairs will never actually break.

16a. [Individual] Some administrative changes will be taking place next year, and Jonathan Phillips is thrilled that he will be moving over into a new and better building.

16b. [Group] Some administrative changes will be taking place next year, and the Symphony Orchestra is thrilled that it will be moving over into a new and better building.

16c. [Control] Some administrative changes will be taking place next year, and the latest memo says that the Symphony Orchestra will soon be moving over into a new and better building.

17a. [Individual] The new instruments just arrived on Tuesday morning. Jonathan Phillips was disappointed that the new violins so quickly started going out of tune.

17b. [Group] The new instruments just arrived on Tuesday morning. The Symphony Orchestra was disappointed that the new violins so quickly started going out of tune.

17c. [Control] The new instruments just arrived on Tuesday morning. But the very next day, the computer system already showed that the violins were starting to go out of tune.

18a. [Individual] A new marketing firm had just been hired. When the spreadsheet showed only seventeen tickets sold, Jonathan Phillips began to regret his decision.

18b. [Group] A new marketing firm had just been hired. When the spreadsheet showed only seventeen tickets sold, the Symphony Orchestra began to regret its decision.

18c. [Control] A new marketing firm had just been hired. When the spreadsheet showed only seventeen tickets sold, the computer initiated its 'huge marketing mistake' algorithm.

Spontaneous Theory-of-Mind Task

1. It can be difficult to compete with today's indie rock scene. Would the Symphony Orchestra [Jonathan Phillips] be more likely to (a) compose symphonic versions of indie tunes or (b) just focus on the classical repertoire?

2. A recent report declared the orchestra to be one of the worst in the nation. Would the Symphony Orchestra [Jonathan Phillips] be more likely to (a) fire the conductor or (b) decrease everyone's salary?

3. The best available conductor is extremely ugly. Would the Symphony Orchestra [Jonathan Phillips] be more likely to (a) hire the best conductor or (b) find someone more physically attractive?

4. There are only two audience members in the entire concert hall. Would the Symphony Orchestra [Jonathan Phillips] be more likely to (a) cancel the concert or (b) just play anyway?

5. It's time to create a poster for the new opera. Would the Symphony Orchestra [Jonathan Phillips] be more likely to (a) play up the sexual tension between the characters or (b) focus instead on its historical significance?

6. The first violinist is terribly ill. Would the Symphony Orchestra [Jonathan Phillips] be more likely to (a) make him play a number of concerts anyway or (b) give him some time off to recover?

7. Studies show that bassoonists play better while drunk. Would the Symphony Orchestra [Jonathan Phillips] be more likely to (a) allow bassoonists to drink before concerts or (b) prohibit drinking anyway?

8. Someone snuck into the concert hall to listen to a symphony. Would the Symphony Orchestra [Jonathan Phillips] be more likely to (a) report him to the police or (b) just let him go?

Set 8: NASA

Directed Theory-of-Mind Task

1a. [Individual] No extraterrestrial intelligence had been found so far. Yet Herbert Maclean believed that an encounter with intelligent life from outer space was just around the corner.

1b. [Group] No extraterrestrial intelligence had been found so far. Yet NASA believed that an encounter with intelligent life from outer space was just around the corner.

1c. [Control] No extraterrestrial intelligence had been found so far. Yet mathematical models suggested that an encounter with intelligent life from outer space was just around the corner.

2a. [Individual] Astronomers were required to eat specific foods at specific times. Herbert Maclean thought that they should always eat marmite on Thursday afternoons.

2b. [Group] Astronomers were required to eat specific foods at specific times. NASA thought that they should always to eat marmite on Thursday afternoons.

2c. [Control] Astronomers were required to eat specific foods at specific times. In particular, the official schedule stated that they had to eat marmite on Thursday afternoons.

3a. [Individual] Recent voyages had sent gerbils up on the space shuttle along with the humans, but Herbert Maclean figured that gazelles might be a bit too large to send on such a journey.

3b. [Group] Recent voyages had sent gerbils up on the space shuttle along with the humans, but NASA figured that gazelles might be a bit too large to send on such a journey.

3c. [Control] Recent voyages had sent gerbils up on the space shuttle along with the humans, but computer modeling showed that gazelles would be too large to send on such a journey.

4a. [Individual] Many expensive research projects have failed, but Herbert Maclean truly believes that his latest initiative will turn out to be well worth the investment.

4b. [Group] Many expensive research projects have failed, but NASA truly believes that its latest initiative will turn out to be well worth the investment.

4c. [Control] Many expensive research projects have failed, but economic analyses indicate that this latest initiative will turn out to be worth the investment.

5a. [Individual] The new program might cost more than ten trillion dollars. Yet Herbert Maclean believes that space exploration is the only way to escape our growing environmental problems.

5b. [Group] The new program might cost more than ten trillion dollars. Yet NASA believes that space exploration is the only way to escape our growing environmental problems.

5c. [Control] The new program might cost more than ten trillion dollars. Yet some theories suggest that space exploration is the only way to escape our growing environmental problems.

6a. [Individual] Some measures suggest that the most important research on this topic was done in the 1970's, but Herbert Maclean believes that his best work was done just in the past few years.

6b. [Group] Some measures suggest that the most important research on this topic was done in the 1970's, but NASA believes that its best work was done just in the past few years.

6c. [Control] Some measures suggest that the most important research on this topic was done in the 1970's, but other analyses indicate that the best work was done just in the past few years.

7a. [Individual] The key to the whole thing is to get Congress to pass a new funding bill. Herbert Maclean is completely certain that his new PowerPoint presentation will get that bill passed.

7b. [Group] The key to the whole thing is to get Congress to pass a new funding bill. NASA is completely certain that its new PowerPoint presentation will get that bill passed.

7c. [Control] The key to the whole thing is to get Congress to pass a new funding bill. Perhaps NASA's new PowerPoint presentation will somehow help to get that bill passed.

8a. [Individual] Earlier studies showed that astronauts suffered severe health problems during space voyages, but Herbert Maclean thinks that his new Red Bull energy drink will fix everything.

8b. [Group] Earlier studies showed that astronauts suffered severe health problems during space voyages, but NASA thinks that their new Red Bull energy drink will fix everything.

8a. [Control] Earlier studies showed that astronauts suffered severe health problems during space voyages. It is clear that the new Red Bull energy drinks won't solve this problem.

9a. [Individual] If it is important to study the possibility of colonizing other planets, Herbert Maclean thinks a good first step would be to see whether we can grow slime molds on the moon.

9b. [Group] If it is important to study the possibility of colonizing other planets. NASA thinks a good first step would be to see whether we can grow slime molds on the moon.

9c. [Control] If it is important to study the possibility of colonizing other planets at this point, a good first step would be to see whether we can grow slime molds on the moon.

10a. [Individual] The next shuttle launch will take place in July. Herbert Maclean is thrilled that this new launch will include some excellent cuisine, including a new type of cheese omelette.

10b. [Group] The next shuttle launch will take place in July. NASA is thrilled that this new launch will include some excellent cuisine, including a new type of cheese omelette.

10c. [Control] The next shuttle launch will take place in July. The annual report indicates that this new launch will include some excellent cuisine, including a new type of cheese omelette.

11a. [Individual] Although it was simply the only way that the project could be completed in a cost-effective manner, Herbert Maclean regrets that all of those forests had to be destroyed.

11b. [Group] Although it was simply the only way that the project could be completed in a cost-effective manner, NASA regrets that all of those forests had to be destroyed

11c. [Control] The reports showed that a number of forests ended up being destroyed, but it was simply the only way that the project could be completed in a cost-effective manner.

12a. [Individual] A new pop hit at the top of the charts actually mentions the space shuttle. But Herbert Maclean was disappointed that the song portrayed his last space voyage in such a negative light.

12b. [Group] A new pop hit at the top of the charts actually mentions the space shuttle. But NASA was disappointed that the song portrayed their last space voyage in such a negative light.

12c. [Control] A new pop hit at the top of the charts actually mentions the space shuttle. But the review in Spin magazine says that it portrays the last space voyage in a negative light.

13a. [Individual] The official NASA logo is now on all of its major reports. But Herbert Maclean regrets that he can never seem to get that logo onto network television or any of those HBO shows.

13b. [Group] The official NASA logo is now on all of its major reports. But NASA regrets that it can never seem to get that logo onto network television or any of those HBO shows.

13c. [Control] The official NASA logo is now on all of its major reports. But it definitely seems like that logo has never appeared on network television or any of those HBO shows.

14a. [Individual] The new NASA initiatives will be introduced at the annual meeting. Herbert Maclean is especially excited that he will be starting a blog on space exploration.

14b. [Group] The new NASA initiatives will be introduced at the annual meeting. NASA is especially excited that they will be starting a blog on space exploration.

14a. [Control] The new NASA initiatives will be introduced at the annual meeting. According to the agenda, one of these initiatives will include starting a blog on space exploration.

15a. [Individual] Some had suggested that we might take cute little kittens into outer space, but ever since the 1980's, Herbert Maclean had been deeply opposed to all such publicity stunts.

15b. [Group] Some had suggested that we might take cute little kittens into outer space, but ever since the 1980's, NASA had been deeply opposed to all such publicity stunts.

15c. [Control] Some had suggested that we might take cute little kittens into outer space, but ever since the 1980's, there had been absolutely no use of any such publicity stunts.

16a. [Individual] How can we make people more effective during space flights? Herbert Maclean hoped that he could look into possible benefits from certain recreational drugs.

16b. [Group] How can we make people more effective during space flights? NASA hoped that they could look into possible benefits from certain recreational drugs.

16c. [Control] How can we make people more effective during space flights? Some scientific studies had pointed to possible benefits from certain recreational drugs.

17a. [Individual] The new publicity drive was just about to begin. Herbert Maclean was excited that a new movie was being made about his major accomplishments.

17b. [Group] The new publicity drive was just about to begin. NASA was excited that a new movie was being made about its major accomplishments.

17c. [Control] The new publicity drive was just about to begin. The annual report indicated that a firm would be hired to make a movie about NASA's major accomplishments.

18a. [Individual] Finding all those rare metals for the space shuttle had led to the destruction of several forests. Herbert Maclean deeply regrets the environmental problems he has caused.

18b. [Group] Finding all those rare metals for the space shuttle had led to the destruction of several forests. NASA deeply regrets the environmental problems it has caused.

18c. [Control] Finding all those rare metals for the space shuttle had led to the destruction of several forests. NASA ended up causing some quite serious environmental problems.

Spontaneous Theory-of-Mind task

1. The space shuttle just exploded, killing everyone aboard. Would NASA [Herbert Maclean] be more likely to (a) accept full responsibility for the incident or (b) blame the whole thing on bad luck?

2. There are plans to send an animal on a voyage to Mars. Would NASA [Herbert Maclean] be more likely to (a) send an adorable little kitten or (b) just send a colony of insects?

3. Physicists have suggested that manned space flights have little scientific value. Would NASA [Herbert Maclean] be more likely to (a) respond with careful arguments or (b) personally attack the physicists themselves?

4. A satellite with people onboard has been lost in orbit. Would NASA [Herbert Maclean] be more likely to (a) spend as much as necessary to save the astronauts or (b) put an absolute limit on the project budget?

5. A rebellious group of young astrophysicists has proposed a bold new hypothesis. Would NASA [Herbert Maclean] be more likely to (a) test the new hypothesis or (b) just continue business as usual?

6. It’s time for a new spacesuit design. Would NASA [Herbert Maclean] be more likely to (a) hire a fashion consultant or (b) simply use whichever design would be most effective?

7. There is a new program afoot to educate children about space. Would NASA [Herbert Maclean] be more likely to (a) put together a video with cute animated characters or (b) just present the scientific facts?

8. A major error was uncovered in the executive compensation plan. Would NASA [Herbert Maclean] be more likely to (a) hire an independent auditor or (b) shred all of the documents immediately?
