# Supplementary material for: The Neural Bases of Directed and Spontaneous Mental State Attributions to Group Agents
Source: PLoS One. 2014 Aug 20;9(8):e105341. doi: 10.1371/journal.pone.0105341 (PMC4139375; doi:10.1371/journal.pone.0105341)
Supplement: Table S1 — Data from Experiment 1. Individual subject responses for each vignette. Condition 1 = ‘any member’; 2 = ‘each member’; 3 = ‘group’. (PDF) [file pone.0105341.s003.pdf]

Order	Cond	Cactus	National	Refresh	Shady	Shuttle	DissRed	CoAssoc	NAPCM	M/F	Age	Group	Members
2.00	1.00	5.00	4.00	1.00	6.00	2.00	6.00	4.00	7.00	1.00	20.00	3.00	5.75
3.00	1.00	1.00	7.00	1.00	7.00	2.00	6.00	4.00	7.00	0.00	21.00	2.00	6.75
4.00	1.00	7.00	5.00	1.00	7.00	2.00	7.00	1.00	7.00	1.00	18.00	2.75	6.50
5.00	1.00	6.00	2.00	3.00	6.00	3.00	7.00	2.00	2.00	0.00	19.00	3.50	4.25
6.00	1.00	2.00	6.00	3.00	7.00	4.00	7.00	5.00	6.00	0.00	18.00	3.50	6.50
7.00	1.00	7.00	4.00	4.00	7.00	1.00	7.00	2.00	5.00	0.00	21.00	3.50	5.75
8.00	1.00	2.00	6.00	5.00	7.00	7.00	4.00	4.00	7.00	0.00	20.00	4.50	6.00
2.00	1.00	1.00	1.00	1.00	1.00	4.00	7.00	1.00	1.00	0.00	18.00	1.75	2.50
1.00	1.00	7.00	6.00	6.00	5.00	1.00	6.00	7.00	6.00	1.00	19.00	5.25	5.75
3.00	1.00	4.00	4.00	7.00	7.00	1.00	7.00	7.00	4.00	0.00	19.00	4.75	5.50
4.00	1.00	1.00	7.00	1.00	5.00	2.00	7.00	1.00	7.00	0.00	18.00	1.25	6.50
5.00	1.00	4.00	5.00	4.00	7.00	4.00	7.00	4.00	7.00	0.00	20.00	4.00	6.50
6.00	1.00	6.00	3.00	2.00	5.00	2.00	5.00	4.00	7.00	1.00	18.00	3.50	5.00
7.00	1.00	1.00	7.00	1.00	7.00	7.00	7.00	1.00	7.00	0.00	51.00	2.50	7.00
8.00	1.00	1.00	6.00	1.00	7.00	5.00	7.00	2.00	7.00	0.00	21.00	2.25	6.75
1.00	1.00	6.00	7.00	2.00	5.00	7.00	7.00	4.00	7.00	1.00	19.00	4.75	6.50
2.00	1.00	1.00	7.00	4.00	4.00	3.00	7.00	1.00	7.00	0.00	21.00	2.25	6.25
3.00	1.00	1.00	7.00	1.00	7.00	1.00	7.00	5.00	7.00	1.00	19.00	2.00	7.00
4.00	1.00	3.00	3.00	4.00	5.00	2.00	6.00	2.00	5.00	1.00	19.00	2.75	4.75
3.00	1.00	2.00	6.00	4.00	7.00	1.00	7.00	5.00	7.00	0.00	18.00	3.00	6.75
4.00	1.00	1.00	7.00	6.00	7.00	1.00	7.00	1.00	7.00	1.00	20.00	2.25	7.00
3.00	1.00	1.00	7.00	1.00	7.00	2.00	7.00	1.00	7.00	0.00	20.00	1.25	7.00
2.00	1.00	1.00	7.00	7.00	7.00	1.00	7.00	4.00	4.00	0.00	18.00	3.25	6.25
4.00	1.00	1.00	7.00	4.00	7.00	6.00	7.00	1.00	7.00	0.00	20.00	3.00	7.00
5.00	1.00	1.00	7.00	7.00	7.00	1.00	7.00	7.00	7.00	1.00	18.00	4.00	7.00
3.00	1.00	2.00	6.00	3.00	6.00	4.00	5.00	5.00	3.00	0.00	26.00	3.50	5.00
8.00	1.00	4.00	6.00	2.00	6.00	2.00	6.00	3.00	5.00	1.00	18.00	2.75	5.75
7.00	1.00	4.00	7.00	1.00	7.00	4.00	7.00	5.00	7.00	0.00	18.00	3.50	7.00
1.00	1.00	1.00	6.00	1.00	7.00	2.00	5.00	3.00	7.00	0.00	21.00	1.75	6.25
1.00	1.00	4.00	7.00	4.00	5.00	1.00	6.00	4.00	4.00	1.00	20.00	3.25	5.50
6.00	1.00	3.00	7.00	4.00	7.00	1.00	7.00	1.00	6.00	0.00	19.00	2.25	6.75
2.00	1.00	6.00	7.00	1.00	7.00	4.00	7.00	7.00	7.00	1.00	20.00	4.50	7.00
7.00	1.00	1.00	7.00	1.00	4.00	7.00	7.00	1.00	7.00	0.00	19.00	2.50	6.25
8.00	1.00	1.00	1.00	1.00	7.00	4.00	7.00	1.00	7.00	0.00	22.00	1.75	5.50
6.00	1.00	2.00	6.00	6.00	7.00	5.00	6.00	2.00	7.00	0.00	20.00	3.75	6.50
4.00	1.00	1.00	7.00	1.00	7.00	1.00	7.00	1.00	7.00	0.00		1.00	7.00
5.00	1.00	1.00	4.00	4.00	4.00	7.00	7.00	1.00	4.00	0.00	20.00	3.25	4.75
3.00	1.00	3.00	6.00	2.00	6.00	6.00	7.00	4.00	7.00	1.00	20.00	3.75	6.50
1.00	1.00	1.00	5.00	1.00	7.00	2.00	7.00	1.00	7.00	0.00	18.00	1.25	6.50
2.00	1.00	5.00	4.00	5.00	5.00	2.00	5.00	4.00	4.00	1.00	18.00	4.00	4.50
1.00	2.00	1.00	1.00	7.00	1.00	7.00	2.00	4.00	6.00	1.00	18.00	4.75	2.50
3.00	2.00	7.00	7.00	1.00	1.00	7.00	7.00	1.00	1.00	1.00	19.00	4.00	4.00
2.00	2.00	7.00	6.00	5.00	1.00	6.00	6.00	1.00	6.00	0.00	21.00	4.75	4.75
4.00	2.00	7.00	2.00	1.00	1.00	1.00	5.00	6.00	5.00	1.00	19.00	3.75	3.25
5.00	2.00	7.00	1.00	7.00	1.00	7.00	6.00	4.00	2.00	0.00	18.00	6.25	2.50
6.00	2.00	6.00	2.00	5.00	2.00	7.00	1.00	2.00	3.00	1.00	20.00	5.00	2.00
7.00	2.00	4.00	1.00	2.00	1.00	7.00	2.00	1.00	1.00	0.00	18.00	3.50	1.25
8.00	2.00	7.00	7.00	7.00	1.00	7.00	7.00	7.00	5.00	1.00	23.00	7.00	5.00
2.00	2.00	7.00	4.00	7.00	1.00	7.00	5.00	4.00	4.00	1.00	20.00	6.25	3.50
1.00	2.00	6.00	2.00	6.00	2.00	6.00	4.00	2.00	2.00	0.00	18.00	5.00	2.50
3.00	2.00	7.00	1.00	7.00	1.00	7.00	7.00	7.00	1.00	1.00	21.00	7.00	2.50
4.00	2.00	7.00	1.00	1.00	7.00	7.00	7.00	4.00	1.00	0.00	20.00	4.75	4.00
5.00	2.00	4.00	4.00	5.00	1.00	7.00	3.00	3.00	2.00	0.00	18.00	4.75	2.50
6.00	2.00	7.00	3.00	6.00	5.00	7.00	6.00	7.00	6.00	0.00	21.00	6.75	5.00
7.00	2.00	6.00	5.00	5.00	2.00	6.00	6.00	3.00	6.00	0.00	22.00	5.00	4.75
8.00	2.00	5.00	4.00	3.00	3.00	5.00	4.00	5.00	4.00	0.00	21.00	4.50	3.75
1.00	2.00	6.00	3.00	7.00	3.00	7.00	6.00	1.00	4.00	0.00	19.00	5.25	4.00
2.00	2.00	7.00	1.00	6.00	1.00	7.00	7.00	1.00	5.00	0.00	21.00	5.25	3.50
3.00	2.00	1.00	3.00	5.00	2.00	6.00	6.00	1.00	3.00	1.00	19.00	3.25	3.50
4.00	2.00	7.00	7.00	1.00	7.00	7.00	7.00	1.00	7.00	0.00	18.00	4.00	7.00
8.00	2.00	1.00	6.00	6.00	3.00	6.00	7.00	6.00	1.00	1.00	19.00	4.75	4.25
5.00	2.00	7.00	1.00	7.00	1.00	7.00	1.00	7.00	1.00	0.00	30.00	7.00	1.00
6.00	2.00	2.00	7.00	4.00	6.00	3.00	6.00	2.00	7.00	0.00	18.00	2.75	6.50
4.00	2.00	6.00	2.00	6.00	3.00	7.00	6.00	1.00	2.00	1.00	21.00	5.00	3.25
3.00	2.00	5.00	6.00	5.00	7.00	4.00	7.00	4.00	7.00	0.00	18.00	4.50	6.75

2.00	2.00	4.00	4.00	7.00	1.00	7.00	6.00	2.00	1.00	0.00	22.00	5.00	3.00
1.00	2.00	1.00	1.00	7.00	1.00	7.00	7.00	4.00	1.00	1.00	20.00	4.75	2.50
4.00	2.00	7.00	1.00	7.00	1.00	7.00	7.00	6.00	1.00	0.00	19.00	6.75	2.50
3.00	2.00	7.00	1.00	7.00	7.00	7.00	7.00	7.00	1.00	1.00	18.00	7.00	4.00
1.00	2.00	7.00	6.00	2.00	2.00	7.00	6.00	4.00	2.00	1.00	19.00	5.00	4.00
2.00	2.00	4.00	1.00	7.00	1.00	7.00	7.00	1.00	1.00	0.00	20.00	4.75	2.50
8.00	2.00	7.00	5.00	5.00	3.00	7.00	6.00	2.00	6.00	0.00	19.00	5.25	5.00
7.00	2.00	5.00	1.00	5.00	1.00	7.00		7.00	1.00	0.00	21.00	6.00	
5.00	2.00	7.00	1.00	7.00	1.00	7.00	4.00	1.00	4.00	0.00	19.00	5.50	2.50
4.00	2.00	7.00	1.00	7.00	1.00	7.00	2.00	1.00	1.00	0.00	25.00	5.50	1.25
6.00	2.00	7.00	1.00	5.00	1.00	7.00	7.00	6.00	2.00	1.00	20.00	6.25	2.75
3.00	2.00	3.00	6.00	7.00	1.00	7.00	7.00	4.00	4.00	0.00	20.00	5.25	4.50
2.00	2.00	6.00	6.00	2.00	2.00	6.00	1.00	6.00	5.00	0.00	43.00	5.00	3.50
1.00	2.00	2.00	5.00	1.00	2.00	7.00	6.00	3.00	5.00	1.00	22.00	3.25	4.50
7.00	2.00	7.00	1.00	7.00	7.00	7.00	7.00	1.00	1.00	0.00	21.00	5.50	4.00
1.00	3.00	1.00	1.00	1.00	7.00	1.00	7.00	1.00	1.00	0.00	19.00	1.00	4.00
2.00	3.00	1.00	4.00	1.00	7.00	1.00	6.00	1.00	6.00	0.00	22.00	1.00	5.75
3.00	3.00	5.00	7.00	1.00	1.00	5.00	5.00	7.00	7.00	0.00	19.00	4.50	5.00
5.00	3.00	1.00	4.00	1.00	4.00	2.00	7.00	4.00	7.00	0.00	20.00	2.00	5.50
4.00	3.00	1.00	7.00	1.00	7.00	4.00	4.00	1.00	7.00	0.00	21.00	1.75	6.25
6.00	3.00	5.00	5.00	1.00	7.00	1.00	7.00	1.00	2.00	0.00	21.00	2.00	5.25
7.00	3.00	1.00	3.00	1.00	1.00	3.00	5.00	2.00	4.00	0.00	20.00	1.75	3.25
8.00	3.00	1.00	7.00	1.00	7.00	1.00	4.00	1.00	7.00	0.00	20.00	1.00	6.25
2.00	3.00	1.00	7.00	1.00	1.00	1.00	7.00	1.00	1.00	1.00	18.00	1.00	4.00
3.00	3.00	1.00	1.00	1.00	4.00	3.00	6.00	1.00	1.00	1.00	19.00	1.50	3.00
1.00	3.00	1.00	4.00	1.00	2.00	1.00	7.00	1.00	7.00	0.00	18.00	1.00	5.00
6.00	3.00	1.00	5.00	1.00	7.00	1.00	4.00	1.00	1.00	0.00	20.00	1.00	4.25
7.00	3.00	7.00	4.00	2.00	6.00	1.00	1.00	2.00	6.00	1.00	21.00	3.00	4.25
8.00	3.00	1.00	7.00	1.00	7.00	2.00	4.00	5.00	7.00	0.00	18.00	2.25	6.25
1.00	3.00	6.00	1.00	1.00	1.00	1.00	7.00	1.00	7.00	0.00	19.00	2.25	4.00
2.00	3.00	2.00	2.00	1.00	7.00	2.00	6.00	3.00	7.00	0.00	20.00	2.00	5.50
3.00	3.00	1.00	4.00	4.00	7.00	1.00	7.00	1.00	7.00	0.00	18.00	1.75	6.25
4.00	3.00	1.00	7.00	1.00	7.00	1.00	7.00	1.00	7.00	1.00	20.00	1.00	7.00
4.00	3.00	1.00	7.00	1.00	7.00	1.00	7.00	1.00	1.00	0.00	18.00	1.00	5.50
2.00	3.00	4.00	4.00	4.00	6.00	6.00	4.00	1.00	7.00	1.00	24.00	3.75	5.25
3.00	3.00	1.00	7.00	1.00	7.00	2.00	6.00	4.00	6.00	0.00	19.00	2.00	6.50
1.00	3.00	2.00	3.00	1.00	7.00	1.00	6.00	2.00	7.00	0.00	19.00	1.50	5.75
8.00	3.00	1.00	4.00	1.00	7.00	1.00	1.00	4.00	7.00	0.00	18.00	1.75	4.75
7.00	3.00	1.00	7.00	1.00	7.00	1.00	7.00	1.00	7.00	0.00	20.00	1.00	7.00
6.00	3.00	3.00	6.00	6.00	6.00	2.00	5.00	2.00	6.00	0.00	19.00	3.25	5.75
4.00	3.00	4.00	6.00	2.00	6.00	2.00	6.00		6.00	1.00	20.00		6.00
1.00	3.00	1.00	1.00	2.00	1.00	1.00	1.00	1.00	4.00	1.00	21.00	1.25	1.75
8.00	3.00	2.00	5.00	2.00	7.00	1.00	7.00	2.00	6.00	0.00	20.00	1.75	6.25
3.00	3.00	6.00	4.00	1.00	7.00	1.00	7.00	3.00	4.00	0.00	21.00	2.75	5.50
2.00	3.00	1.00	7.00	1.00	7.00	1.00	7.00	2.00	7.00	1.00	19.00	1.25	7.00
4.00	3.00	1.00	4.00	2.00	4.00	2.00	6.00	5.00	6.00	0.00	19.00	2.50	5.00
7.00	3.00	1.00	5.00	1.00	7.00	1.00	4.00	4.00	6.00	0.00	18.00	1.75	5.50
5.00	3.00	1.00	7.00	1.00	7.00	2.00	6.00	1.00	7.00	0.00	21.00	1.25	6.75
6.00	3.00	1.00	5.00	1.00	5.00	1.00	6.00	1.00	7.00	0.00	20.00	1.00	5.75
3.00	3.00	1.00	1.00	1.00	2.00	1.00	6.00	1.00	1.00	0.00	22.00	1.00	2.50
4.00	3.00	7.00	1.00	7.00	4.00	1.00	7.00	7.00	1.00	1.00	54.00	5.50	3.25
2.00	3.00	1.00	7.00	1.00	7.00	3.00	7.00	1.00	7.00	1.00	19.00	1.50	7.00
1.00	3.00	4.00	6.00	4.00	7.00	1.00	6.00	4.00	7.00	0.00	19.00	3.25	6.50
