# Supplementary material for: The Neural Bases of Directed and Spontaneous Mental State Attributions to Group Agents
Source: PLoS One. 2014 Aug 20;9(8):e105341. doi: 10.1371/journal.pone.0105341 (PMC4139375; doi:10.1371/journal.pone.0105341)
Supplement: Table S2 — Data from Experiment 2. Mean percent signal change (PSC) for each subject in each condition of the directed and spontaneous theory-of-mind tasks in regions identified by the theory-of-mind localizer. (PDF) [file pone.0105341.s004.pdf]

Directed Theory-of-Mind Task

**TPJ**			**Precuneus**			**mPFC**		
Individual	*Group*	*Control*	*Individual*	*Group*	*Control*	*Individual*	*Group*	*Control*
0.3632	0.3268	0.1152	0.5702	0.5336	0.3393	0.2585	0.2450	0.1549
-0.1229	-0.0710	-0.1570	-0.0020	0.0198	-0.2396	-0.0773	0.0090	-0.0302
0.1921	0.1890	0.1691	0.0971	-0.1115	-0.2876	0.1675	0.0498	0.2815
-0.2844	-0.1748	-0.2266	-0.3802	-0.2130	-0.4893	0.0275	0.3097	0.0912
-0.0251	0.0487	0.0754	0.6061	0.2721	0.1064	0.3755	0.4661	0.3032
0.1982	0.0977	-0.0194	0.4552	0.2710	0.0357	0.2939	0.2278	0.2123
-0.7475	-0.4405	-0.8279	-0.1071	-0.2152	-0.1967			
-0.2750	-0.1214	-0.1917	-0.0493	0.0093	-0.1082	0.3312	0.3402	0.2192
0.3897	0.3492	0.2743	0.1708	0.1255	-0.0282	-0.0159	-0.0164	0.1301
0.0230	-0.2197	-0.1901	0.2174	0.2507	0.2060	-0.0061	-0.1983	-0.1585
0.1618	0.1700	-0.0057	-0.0342	-0.0806	-0.1844			
-0.2971	-0.4746	-0.4001	0.0179	-0.1497	-0.0460	-0.2766	-0.4436	-0.7599
-0.1812	-0.2826	-0.1951	0.2275	0.0150	-0.1623	0.6417	0.5170	0.5889
-0.1806	-0.1598	-0.1989	0.2247	0.1903	-0.1479	0.0582	0.1062	-0.1770
-0.1214	-0.0306	0.0496	0.0825	0.1151	0.0170	0.1659	0.1108	0.0298
0.0833	0.0820	-0.1106	0.1135	0.1625	-0.0051	0.1771	0.2445	0.0602
0.0730	0.0254	-0.0884	-0.2083	0.0459	-0.2388	-0.0879	0.4415	-0.1991
0.0615	-0.0281	-0.1490	0.0971	0.0201	-0.2060	0.2192	0.2396	0.2365
-0.3747	-0.3979	-0.3146	0.0643	-0.0151	-0.1015	0.1484	0.2479	0.0577

Spontaneous Theory-of-Mind Task

**TPJ**		**Precuneus**		**mPFC**	
Individual	*Group*	*Individual*	*Group*	*Individual*	*Group*
0.0039	0.1481	0.4783	0.5516	0.1963	0.3127
0.0295	-0.0734	-0.0181	-0.0350	0.0111	-0.1128
0.2591	0.1012	0.3519	0.5408	-0.0022	-0.0147
-0.2495	-0.2645	0.3779	0.2293	0.3793	0.2177
-0.0309	0.0043	0.8080	0.7540	0.5144	0.8645
-0.0477	0.0056	0.4801	0.3218	0.1373	0.1189
-0.7536	-0.7819	-0.0349	-0.0869		
-0.3392	-0.3255	0.0404	-0.0508	0.4237	0.2694
0.6815	0.6504	0.3864	0.3132	0.0359	0.1137
0.4192	0.2849	0.3555	0.3488	0.0487	0.0003
0.3669	0.3361	0.0290	0.0778		
-0.0175	0.0005	0.0851	0.0798	-0.5277	-0.3669
0.1470	0.1821	0.3303	0.3639	0.6083	1.2578
-0.2565	-0.2296	0.4173	0.3068	-0.0833	-0.0805
0.0525	0.0204	-0.0956	-0.1046	0.2756	-0.2166
0.2112	0.1953	0.2274	0.2271	0.3944	0.4419
-0.3238	-0.2655	0.2619	0.1686	0.1561	0.2173
0.0799	-0.0513	0.4788	0.2651	0.6798	0.5839
-0.3102	-0.2981	0.0908	0.1123		
